# High-entropy perovskite halides: design principles, properties, and emerging applications

**DOI:** 10.1039/d6sc04531f

**Published:** 2026-08-25

**Authors:** Ratiani Levan, Seung Jin Lee, Toshali Bhoyar, Kwangyeol Lee

**Affiliations:** a Department of Chemistry and Center for Multielement Nanoparticle Tectonics, Korea University Seoul 02841 Republic of Korea kylee1@korea.ac.kr

## Abstract

High-entropy perovskite halides are emerging as a compelling extension of compositional engineering for overcoming the persistent instability and performance limitations of conventional metal perovskite halides. By incorporating multiple principal components at sufficiently high concentrations, configurational entropy can act as a thermodynamic stabilizing factor enabling access to compositionally complex phases with modified structural, ionic, and optoelectronic properties. This review examines the design principles, synthesis strategies, and structure–property relationships that define this rapidly developing class of materials. We discuss how entropy-driven compositional complexity influences phase stability, lattice distortion, defect chemistry, ion migration, and band-edge electronic structure, with particular attention to the distinct roles of A-site and B-site disorder. We further summarize current synthetic routes, as well as emerging computational frameworks for screening and guiding the design of high-entropy compositions. Recent advances in light-emitting devices, photovoltaics, photodetection, imaging, sensing and electrochemical applications are then highlighted to illustrate how entropy engineering can improve both functionality and durability. Finally, we identify key unresolved challenges in compositional control, structural characterization, and device integration, and outline future directions for the rational development of high-entropy perovskite halides as robust optoelectronic materials.

## Abstract 2

High-entropy perovskite halides are emerging as a compelling extension of compositional engineering for overcoming the persistent instability and performance limitations of conventional metal perovskite halides.

## Graphical abstract

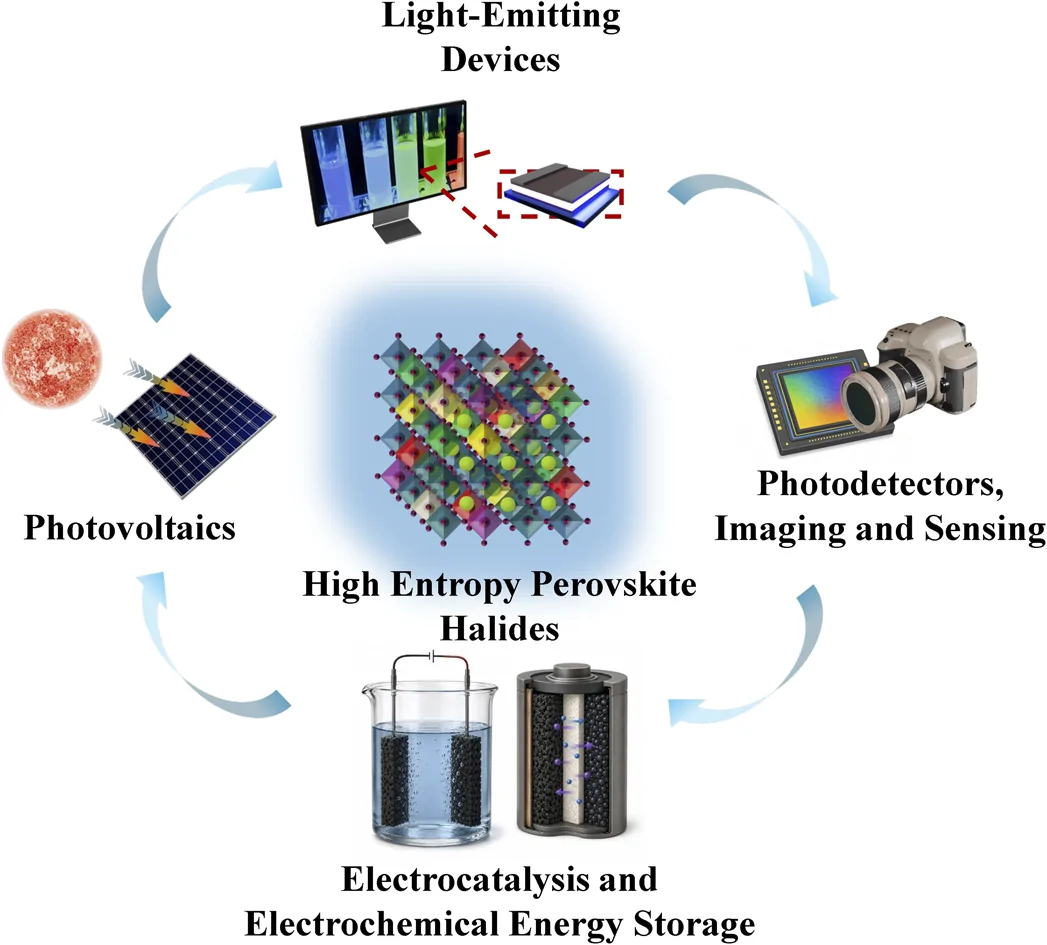

## 1. Introduction

Metal perovskite halides have emerged as a highly versatile semiconductor platform for optoelectronic applications owing to their soft ionic lattices, compositional tunability, defect tolerance, and solution processability. These features enable light emission^1^ and photovoltaic conversion,^2^ positioning metal perovskite halides at the forefront of next-generation light-emitting and solar-energy technologies. Recent device performance highlights this progress, with perovskite light-emitting diodes (PeLEDs) achieving external quantum efficiencies (EQEs) of 26.4% for blue, 28.9% for green, and 28.7% for red emission^3^ and perovskite solar cells (PSCs) exceeding 26% power conversion efficiency (PCE).^4^

However, the same lattice softness and ionic mobility that support high performance also make perovskite halides vulnerable to phase instability, ion migration, local compositional inhomogeneity, and environmental degradation.^5,6^ These limitations pose a major challenge for practical optoelectronic applications, where both compositional engineering and long-term operational stability are required. In PeLEDs, blue and deep-blue emission is particularly challenging because wide-bandgap compositions usually rely on Cl-rich, mixed-halide, or strongly quantum-confined systems that are prone to halide segregation, defect formation, and spectral drift under bias.^5,7^ In PSCs, mixed-cation and mixed-halide absorbers have improved efficiency and stability, but still suffer from photoinduced phase segregation, non-radiative recombination, and long-term degradation.^8–10^ Although conventional compositional engineering has improved efficiency and partially enhanced stability, it has not fully resolved the persistent trade-off between high device performance and long-term operational durability.

To overcome these limitations, extensive efforts have been devoted to improving the stability of perovskite halide devices through strategies that extend beyond conventional compositional engineering.^11^ Approaches based on ligand and surface engineering have been developed to passivate defects and suppress non-radiative recombination,^12^ while additive engineering has been widely employed to regulate crystallization and partially immobilize mobile ions.^11^ In parallel, dimensional engineering has introduced layered or quasi-two-dimensional structures that enhance environmental stability by mitigating ion migration and interfacial degradation.^13^ While these approaches have led to notable improvements in device efficiency and operational lifetime, they primarily rely on extrinsic or localized modifications at surfaces and interfaces. As a result, they often fail to fundamentally suppress ion migration, phase segregation, and defect formation within the bulk lattice,^14^ leaving the intrinsic trade-off between efficiency and durability largely unresolved.

High-entropy perovskite halides have recently emerged as an alternative design strategy to address these limitations. Rather than introducing only one or two substituents, high-entropy perovskite halides incorporate multiple species at sufficiently high concentrations for configurational entropy to become an active thermodynamic factor.^15–17^ In this way, compositional complexity can lower Gibbs free energy, expand accessible phase space, and suppress phase separation.^15,18^ This concept is particularly attractive for perovskite halides because their soft ionic frameworks can accommodate broad substitutional disorder, while the resulting local structural variation can influence defect chemistry, lattice distortion, ion migration, and optical properties simultaneously.^17,19^ However, a comprehensive understanding of entropy-driven design in perovskite halides remains incomplete.^20,21^ In particular, the relationships among compositional complexity, local structural disorder,^22^ and ion transport^23^ are not yet fully established, limiting the rational design of high-entropy systems.

Entropy-driven design enables simultaneous improvements in phase stability, suppression of ion migration, and enhanced optoelectronic performance. This approach offers a pathway toward perovskite materials that combine high efficiency with long-term operational stability, which is essential for next-generation display and solar-energy technologies. By addressing key degradation mechanisms at the material level, high-entropy perovskite halides provide a promising route toward the practical implementation of advanced optoelectronic devices.

This review focuses on recent advances in high-entropy perovskite halides and their potential for light-emitting and photovoltaic applications. Distinct from earlier reviews on high-entropy perovskites that addressed broader energy-conversion and energy-storage materials platforms,^24,25^ the present review specifically centres on halide perovskites and on the optoelectronic consequences of entropy engineering in these soft ionic lattices. In this Review, we discuss the structural foundations, synthesis strategies, computational design principles, and emerging applications of high-entropy perovskite halides. Emphasis is placed on how entropy-driven compositional complexity influences phase stability, ion migration, defect chemistry, and device performance in light-emitting, photovoltaic, photodetector, imaging, and sensing platforms. We further identify the major opportunities, current limitations, and unresolved challenges that define this rapidly developing field.

## 2. Fundamentals

### 2.1 Perovskite and perovskite-derived halide structures

**Fig. 1:**
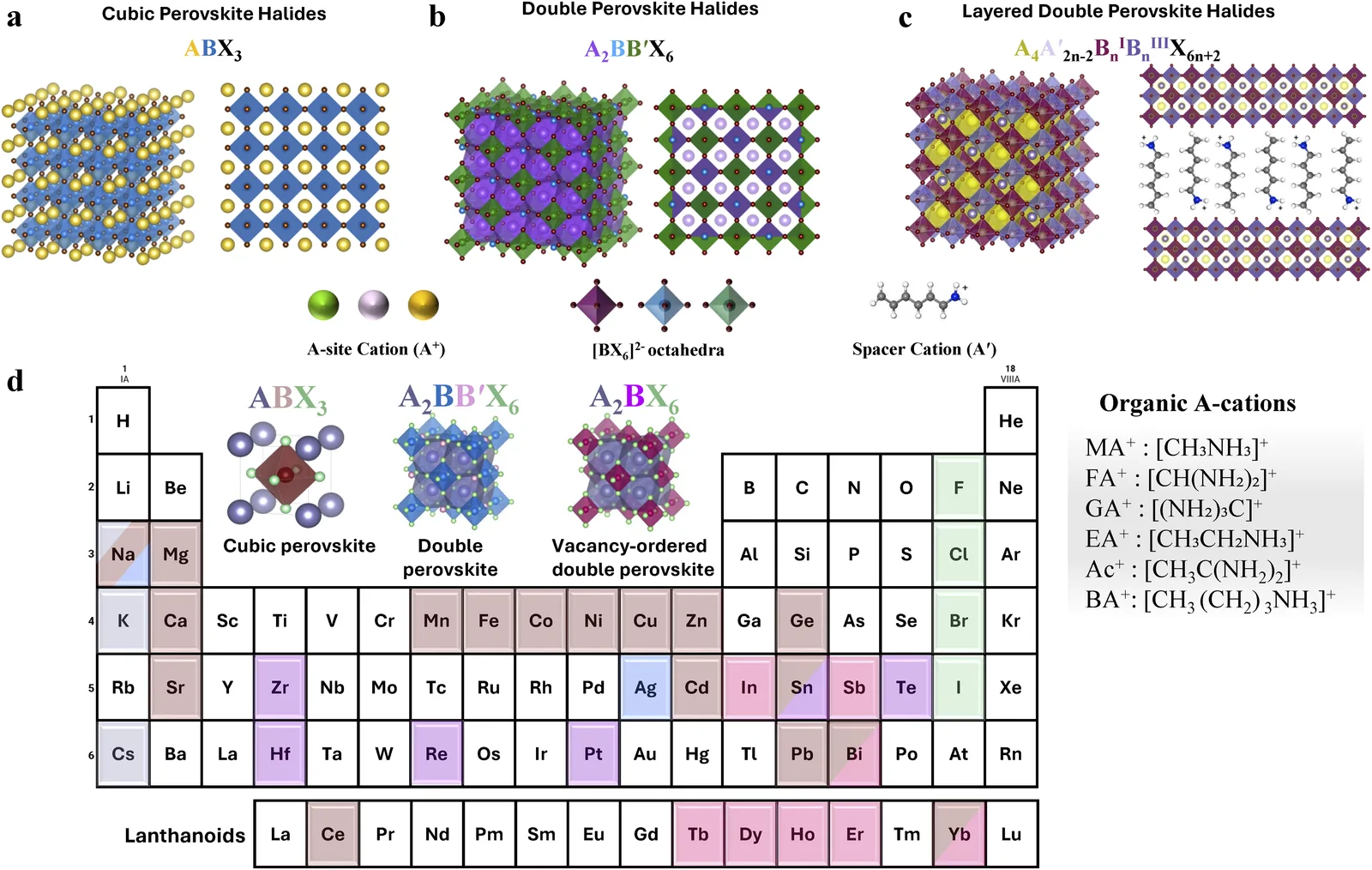
Representative perovskite halide and perovskite-derived halide structures. (a) Cubic perovskite halides, ABX_3_. (b) Double perovskite halides, A_2_BB′X_6_. (c) Layered double perovskite halides, 
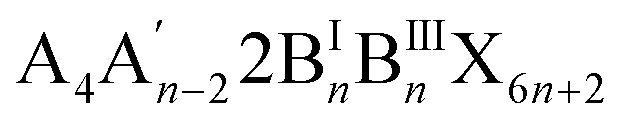
. The bottom legend indicates the A-site cations, representative metal-halide octahedra and organic spacer cation (A′) used in the structural models. (d) Elemental occupancy map of experimentally reported A-, B-, and X-site species in high-entropy perovskite halides, together with protonated amines available for A-sites (MA = methylammonium, FA = formamidinium, GA = guanidinium, EA = ethylammonium, Ac = acetamidinium, and BA = butylammonium).^4,17–19,22,23,31,56,63–65,70,85,86,105,106,108–116^

Perovskite halides are commonly defined by a three-dimensional ABX_3_ lattice built from corner-sharing BX_6_ octahedra, in which the A-site is occupied by a monovalent cation, the B-site by a metal cation, and the X-site by a halide anion. In lead perovskite halides, the A-site is typically filled by alkali-metal or organic ammonium cations (Cs^+^, MA^+^; CH_3_NH_3_^+^), the B-site by Pb^2+^, and the X-site by F^−^, Cl^−^, Br^−^, or I^−^.^26,27^ The structural feasibility of cubic ABX_3_ framework (Fig. 1a) is commonly assessed using the Goldschmidt tolerance factor (*t*) and octahedral factor (*µ*), which describe the geometric fit of the A-site cation and the stability of the BX_6_ octahedron. Reported stability ranges for perovskite halides are approximately 0.813 < *t* < 1.107 and 0.377 < *µ* < 0.895. Although not absolute, these criteria remain useful for discussing how compositional complexity can be introduced without destroying the perovskite framework.^28,29^

Beyond the parent ABX_3_ lattice, the structural landscape relevant to high-entropy perovskite halides also includes double perovskites and other perovskite-derived halides. One important example is the halide double perovskite, A_2_BB′X_6_ (Fig. 1b), in which ordered B-site cations preserve a three-dimensional corner-sharing octahedral framework.^30^ Structural framing provides a basis for understanding how framework connectivity, dimensionality, and vacancy ordering govern lattice stability, ion migration, defect chemistry, charge transport, and optical response. Representative examples of this family include Cs_2_AgBiBr_6_, Cs_2_AgBiCl_6,_ Cs_2_AgInCl_6_, which are frequently discussed as Pb-free or lead-reduced alternatives in halide-perovskite research.^15,31^

A related but distinct structural class is the vacancy-ordered perovskite-derived family, commonly written as A_2_BX_6_. Tai *et al.* described these compounds as derivatives of conventional ABX_3_ perovskites formed by doubling the unit cell along the three axes and removing every other B site cation, so that the remaining B-site species typically adopts 4+ oxidation state to preserve charge neutrality.^32^ Unlike conventional 3D perovskites, these materials contain isolated rather than continuously connected octahedra, which can substantially alter charge transport and exciton behaviour. Representative examples include Cs_2_SnCl_6_ and Cs_2_TeCl_6_ compounds.^33,34^

Layered perovskite halides form another important branch of the perovskite family because dimensional reduction breaks the full three-dimensional connectivity of the inorganic framework. Vargas *et al.* extended the concept of dimensional reduction to halide double perovskites, defining layered double perovskites (LDPs) as 〈100〉-, 〈110〉-, or 〈111〉-oriented depending on the cut through the parent 3D framework. Importantly, they noted that only the 〈100〉 and 〈111〉 families had been experimentally realized at the time of their review, while the 〈110〉 family remained undiscovered.^35^ Representative examples include the 〈100〉-oriented (BA)_4_AgBiBr_8_ and (BA)_2_CsAgBiBr_7_ (Fig. 1c)^36^ derived from Cs_2_AgBiBr_6_, as well as layered vacancy-ordered bismuth halides such as Cs_3_Bi_2_Br_9_,^37^ which contain octahedral BiX_6_ layers and the 〈111〉-oriented Cs_4_CuSb_2_Cl_12_. These layered structures are relevant because reduced dimensionality changes octahedral connectivity, carrier confinement, and structural flexibility relative to 3D perovskites.

For the purposes of this review, perovskite halides are therefore considered within a structurally explicit framework that includes: conventional 3D ABX_3_ perovskites, ordered double perovskites A_2_BB′X_6_, vacancy-ordered A_2_BX_6_ derivatives, and layered perovskite-like halides when they are directly relevant to optoelectronic applications. Such structural framing is important because framework connectivity, dimensionality, and vacancy ordering strongly affect lattice stability, ion migration, defect chemistry, charge transport, and optical response. Establishing these structural classes at the outset is therefore necessary before discussing why compositional engineering, and later high-entropy design, became important in perovskite halides.

### 2.2 Key instabilities and limitations of perovskite halides

Despite their excellent optoelectronic properties, perovskite halides remain fundamentally limited by the structural softness of their ionic lattices. Rakita *et al.* argued that these materials should not be viewed as conventional semiconductors with rigid, static defect populations, but rather as systems with highly dynamic defect landscapes arising from low defect formation energies and limited kinetic barriers to defect migration and reorganization.^6^ The soft lattice of perovskite halides enables their characteristic defect tolerance and solution processability, but also makes them susceptible to structural evolution under light, heat, electric fields, and environmental exposure.

#### 2.2.1 Phase instability

**Fig. 2:**
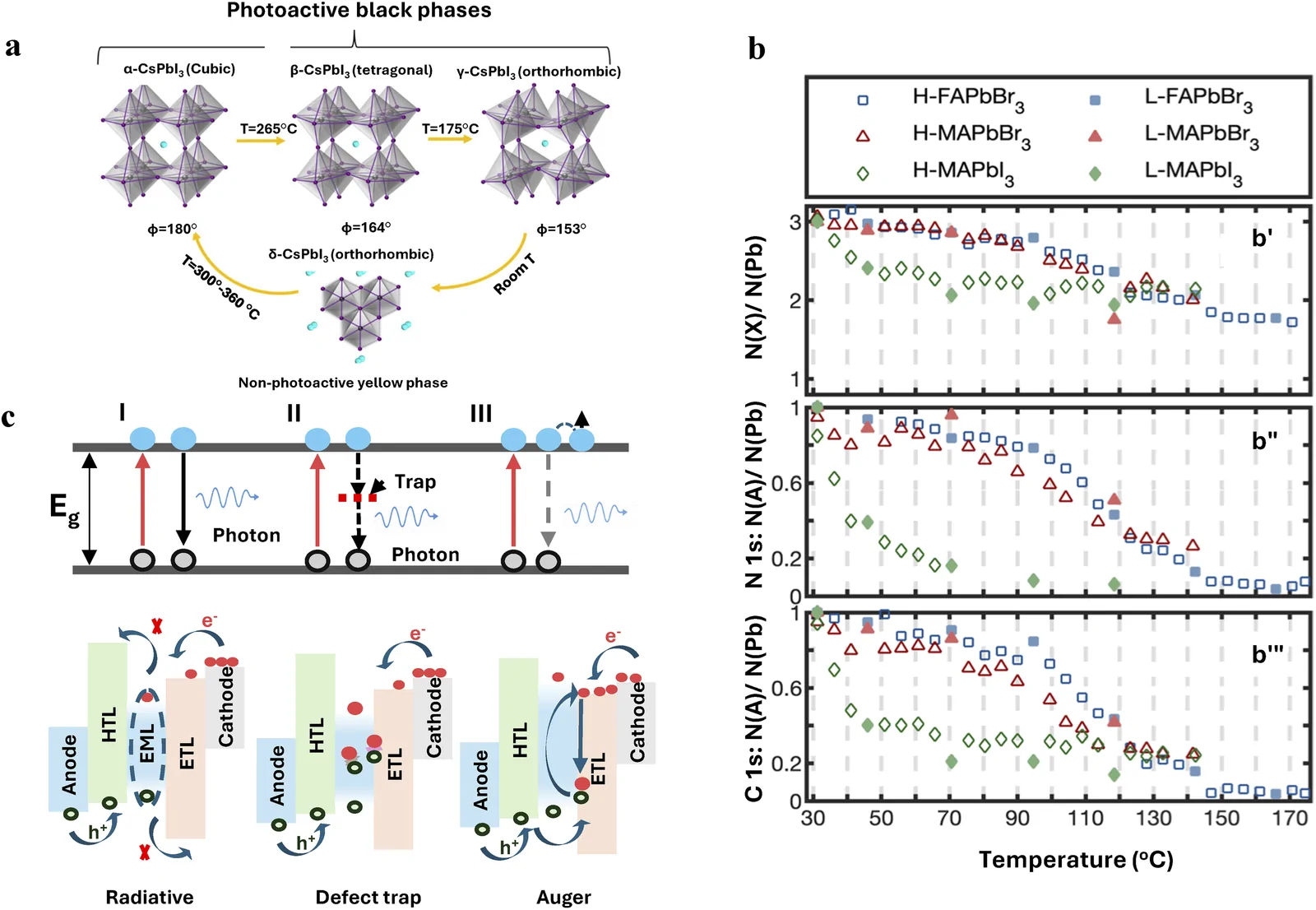
Key instability pathways in perovskite halides. (a) Crystalline structure and polymorphic phase transitions of CsPbI_3_ perovskites. The yellow δ-phase transforms to the black photoactive α-phase at high temperature, whereas distortion of the [PbI_6_]^4−^ octahedra promotes the formation of the lower-symmetry black β- and γ-phases upon cooling. Reproduced with permission.^38^ Copyright 2020, American Chemical Society. (b) Temperature-dependent evolution of core-level intensities relative to Pb 4f, shown as the halide content, N(X)/N(Pb), from I 4d and Br 3d (b′), and the A-site cation content, N(A)/N(Pb), from N 1s (b″) and C 1s (b‴). Initial ratios are set to 3 for halides and 1 for cations. Filled markers denote the low X-ray exposure spot (L), whereas open markers denote the high X-ray exposure spot (H). Reproduced with permission.^39^ Copyright 2022, the Royal Society of Chemistry. (c) Charge-carrier recombination pathways in perovskites and their implications for PeLED operation: radiative recombination, trap-assisted non-radiative recombination, and auger non-radiative recombination, together with representative device-architecture schematics.

Phase instability remains a central limitation in iodide perovskites. Jin *et al.* showed that the photoactive black phases of CsPbI_3_ are intrinsically metastable at room temperature because this composition lies near a critical structural tolerance condition: ideal cubic perovskite halides are typically associated with a Goldschmidt tolerance factor of about 0.9–1.0, whereas CsPbI_3_ has a value of ∼0.8, placing it close to the boundary for structural destabilization.^40^ As a result, the α-phase at high temperature is not readily retained under ambient conditions and can evolve into the black β/γ phases before converting to the non-perovskite δ-phase upon moisture exposure or mild thermal perturbation (Fig. 2a). Complementing this structural picture, Masi *et al.* showed that the A-site cation is not merely a geometric placeholder, but an active regulator of lattice dynamics: changing the A-site modifies Pb–I bond strength, octahedral dynamic disorder, and the barrier to iodide migration (Fig. 2b).^38,39^ Together, these results indicate that phase instability in iodide perovskites arises from the coupling of marginal structural tolerance with soft, dynamically labile lattices, leaving the photoactive phase highly vulnerable to thermal and operational stress.^40,41^

#### 2.2.2 Non-radiative recombination

A second major problem is defect formation and defect-assisted non-radiative recombination. Defect sites in quantum dots and perovskite nanocrystals (NCs) act as trap states that capture charge carriers and suppress radiative recombination, thereby lowering photoluminescence quantum yield and device efficiency. In perovskite NCs specifically, defects are often associated with halide vacancies, dangling bonds, incomplete ligand passivation, and structural defects such as dislocations, vacancies, and stacking faults, all of which can introduce mid-gap states and impair charge dynamics and luminescence.^42–45^ At the device level, these loss pathways appear through the competition between radiative recombination and non-radiative channels such as trap-assisted and auger recombination (Fig. 2c). In this sense, efficiency roll-off in PeLEDs is not a separate issue from defect chemistry, but rather a device-level manifestation of the same non-radiative processes becoming increasingly important at high carrier densities.

#### 2.2.3 Wide bandgap

Wide-bandgap perovskite halides are particularly vulnerable to this behaviour. Duong *et al.* highlighted that perovskites in the 1.7–1.8 eV bandgap range are highly desirable for perovskite-silicon tandem cells, but mixed I/Br compositions in this range are prone to light-induced phase segregation.^46^ They noted that in MAPb(I_1−*x*_Br_*x*_)_3_, a bromide fraction of about *x* ≈ 0.33 is needed to reach a bandgap near 1.75 eV, but the interval 0.3 < *x* < 0.6 is also associated with a miscibility gap and spinodal decomposition. This is directly relevant to both tandem PSCs and blue-emitting PeLEDs, because the compositions needed for higher bandgaps are often the same ones most susceptible to segregation.

#### 2.2.4 Chemical and thermal instability

It should be highlighted that thermal instability can induce phase transitions, ion migration, and lattice distortion, while high temperatures accelerate defect diffusion and non-radiative recombination.^39,47,48^ These effects are consistent with the temperature-dependent degradation evolution (Fig. 2b). In the source study, the halide-to-lead ratio decreased from about 3 to about 2 near 130 °C, while the cation-related signals also declined markedly, indicating that chemical instability upon heating involves concurrent halide and A-site loss. This is highly relevant to PeLEDs, especially blue-emitting devices, where higher operating energies intensify Joule heating and accelerate degradation. More generally, Vargas *et al.* noted that dimensional reduction from 3D perovskites to layered structures was one of the earliest and most successful strategies used to improve material stability, precisely because large, often hydrophobic, organic spacers can improve resistance to water and light, albeit usually at the cost of lower carrier mobility and reduced photovoltaic efficiency.^35^

#### 2.2.5 Efficiency roll-off

Efficiency roll-off remains an important device-level manifestation of these instabilities. Efficiency roll-off in LEDs arises mainly from carrier imbalance, charge saturation, Auger recombination, Joule heating, electron leakage, and electric-field-induced exciton quenching (Fig. 2c).^49–51^ These processes can be viewed as a shift from desirable radiative recombination toward trap-assisted and auger-type non-radiative decay, which progressively limits PeLED efficiency under high-injection conditions. These processes are closely related to the underlying materials problems discussed above, defects, thermal instability, and interfacial charge accumulation, and are especially severe in blue-emitting devices that operate at higher energies.

Taken together, the key limitations of perovskite halides are not isolated issues but coupled consequences of soft ionic frameworks: metastable photoactive phases, halide-vacancy-related defects, ion migration, halide segregation, thermal and chemical degradation, charge-transport imbalance, and non-radiative loss. These recurring problems explain why simple perovskite compositions rarely provide a balance of efficiency, spectral stability, and operational durability required for practical LEDs and solar cells.

### 2.3 From compositional engineering to high-entropy perovskite halides

Conventional compositional engineering in perovskite halides was originally developed as a practical way to improve phase stability, tune bandgap, and suppress degradation, but it does not automatically qualify as high-entropy design. In the broadest sense, perovskite halides already offer an unusually large compositional space because substitution can occur at the A-, B-, and X-sites of the parent ABX_3_ lattice, as well as in related double-perovskite and vacancy-ordered structures.^52^ However, ordinary alloying or multicomponent mixing is usually interpreted in terms of geometric stabilization, defect passivation, or bandgap control, whereas high-entropy design treats configurational entropy itself as an active thermodynamic variable.

The thermodynamic basis of this phenomenon is the Gibbs free-energy relation,

1 Δ*G* = Δ*H* − *T*Δ*S*

where Δ*H* is the enthalpy contribution and Δ*S* is the entropy contribution. In high-entropy materials, increasing configurational entropy (Δ*S*_config_) lowers the −*T*Δ*S* term and can therefore compensate for positive mixing enthalpy, favouring single-phase formation or suppressing phase separation.^53^ The configurational entropy of a material containing multiple species on its cationic and anionic sublattices can be expressed as^25,54^2
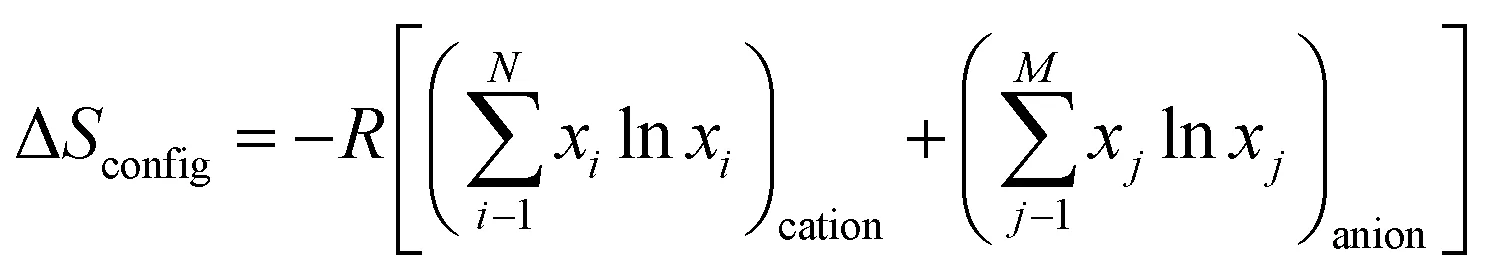
where *R* is the gas constant, while *N* and *M* represent the numbers of chemically distinct species distributed over the cationic and anionic sublattices, respectively. The terms *x*_*i*_ and *x*_*j*_ correspond to the fractional occupancies of those species on each sublattice.

Muzzillo *et al.* applied this concept specifically to perovskite halides and expressed the ideal configurational entropy of mixing for ABX_3_ compounds in a normalized form as *S*/*R*, with *S* denoting the configurational entropy:^20^3
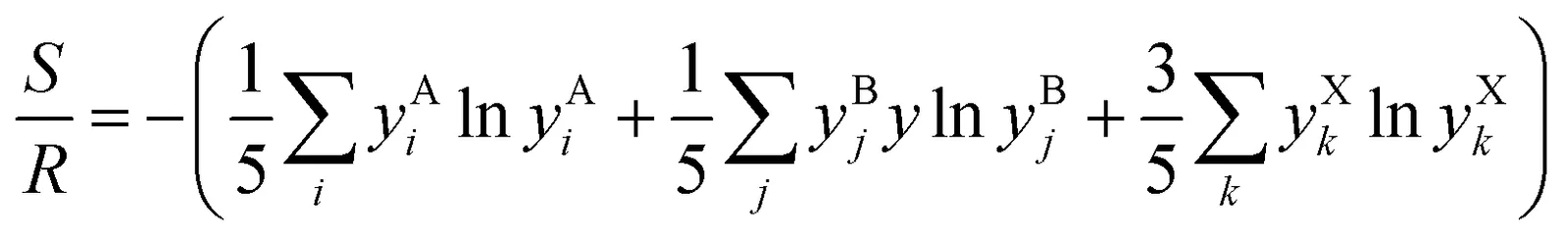
where *y*^A^_*i*_, *y*^B^_*j*_, and *y*^X^_*k*_are the mole fractions on the A, B, and X sublattices, respectively. The corresponding entropy-stabilization (ES) term then becomes,

4 ES = *TS* = *RT*(*S*/*R*)

This formulation is especially useful for perovskite halides because entropy can be introduced selectively at a single crystallographic site or distributed across multiple sublattices.

A key issue is therefore how to distinguish multicomponent, medium-entropy, and high-entropy perovskite halides. In the general literature, true high-entropy materials are usually defined as solids containing five or more principal components in near-equimolar ratios, often corresponding to Δ*S*_config_ values near or above ∼1.5*R* or 1.61*R*.^55,56^ By contrast, systems with fewer principal components are often better classified as low (Δ*S*_config_ ≤ 1.0*R*) or medium-entropy (1.0*R* ≤ Δ*S*_config_ ≤ 1.5*R*) systems. Tai *et al.* explicitly described three- and four-cation Cs_2_BCl_6_ based compositions as medium-entropy perovskites, reserving the stronger high-entropy label for more compositionally complex systems.^57^

In perovskite halides, however, this classification is less straightforward than in single-sublattice alloys because entropy can be introduced at different crystallographic sites. Minussi *et al.* showed that even A-site-only mixing can produce unusually high configurational entropy in hybrid perovskites, using the penta-organic series GA_*x*_FA_*x*_EA_*x*_Ac_*x*_MA_1−4*x*_PbI_3_.^21^ Their results also illustrate an important point: a composition can show significant entropy-driven effects without necessarily satisfying the strictest near-equimolar five-component criterion. Thus, in perovskite halides, the label “high-entropy” should be used carefully and with explicit reference to both site occupancy and configurational entropy.

Current high-entropy perovskite halide research has been pursued mainly through A-site, B-site, or combined A/B-site compositional complexity, whereas X-site high-entropy design is more structurally demanding. More broadly, the accessible X-site chemistry of halide perovskites spans F^−^, Cl^−^, Br^−^, and I^−^, but the practical constraints depend strongly on the specific halide combination. Shen *et al.* noted that much of the field has focused on the A and B sites because octahedral-factor mismatch among [PbF_6_]^4−^, [PbCl_6_]^4−^, [PbBr_6_]^4−^ and [PbI_6_]^4−^ can promote crystal distortion, phase segregation, and halogen migration. However, this route should not be regarded as inaccessible. It is experimentally demonstrated that Cl/Br/I high-entropy metal halide perovskite alloys can be realized through octahedral-factor regulation engineering, which reduces octahedral mismatch and suppresses halide migration to stabilize homogeneous mixed-halide compositions.^23^

For the purposes of this review, high-entropy perovskite halides are therefore considered as perovskite halides or perovskite-derived systems in which multiple principal components are intentionally introduced at sufficiently high concentrations that configurational entropy becomes a meaningful factor in phase formation, structural stabilization, or property evolution. In this sense, high-entropy design should be understood not simply as “more mixing,” but as a more deliberate extension of compositional engineering in which disorder itself becomes a design parameter. The detailed consequences of A-site, B-site, and combined-site entropy engineering will be discussed in later sections.

## 3. Structure–property relationships

The structure–property relationships of high-entropy perovskite halides are strongly site dependent because configurational disorder affects different parts of the lattice in fundamentally different ways. In general, A-site entropy engineering primarily influences organic-cation orientation, rotational freedom, local lattice distortion, and ion migration within the inorganic framework, whereas B-site entropy engineering directly reconstructs the metal-halide electronic network that governs the band-edge structure.^58,59^ Accordingly, this section discusses A-site entropy engineering mainly in terms of lattice stability, ion migration, and environmental response, while B-site entropy engineering is addressed primarily from the perspective of bandgap evolution, excitonic behaviour, and radiative recombination.

### 3.1 A-site entropy engineering and lattice-environment coupling

A-site entropy engineering affects optoelectronic properties primarily through changes in cation arrangement, local lattice distortion, and ion migration rather than through direct reconstruction of the band-edge electronic structure. Because the valence and conduction band edges of perovskite halides are governed primarily by metal-halide orbital interactions, disorder introduced at the A-site primarily modifies orientational dynamics, hydrogen bonding, local lattice distortion, and ionic transport within the perovskite framework. As a result, configurational entropy can substantially alter phase stability and transport behaviour even when the inorganic Pb-X electronic network remains largely preserved.

**Fig. 3:**
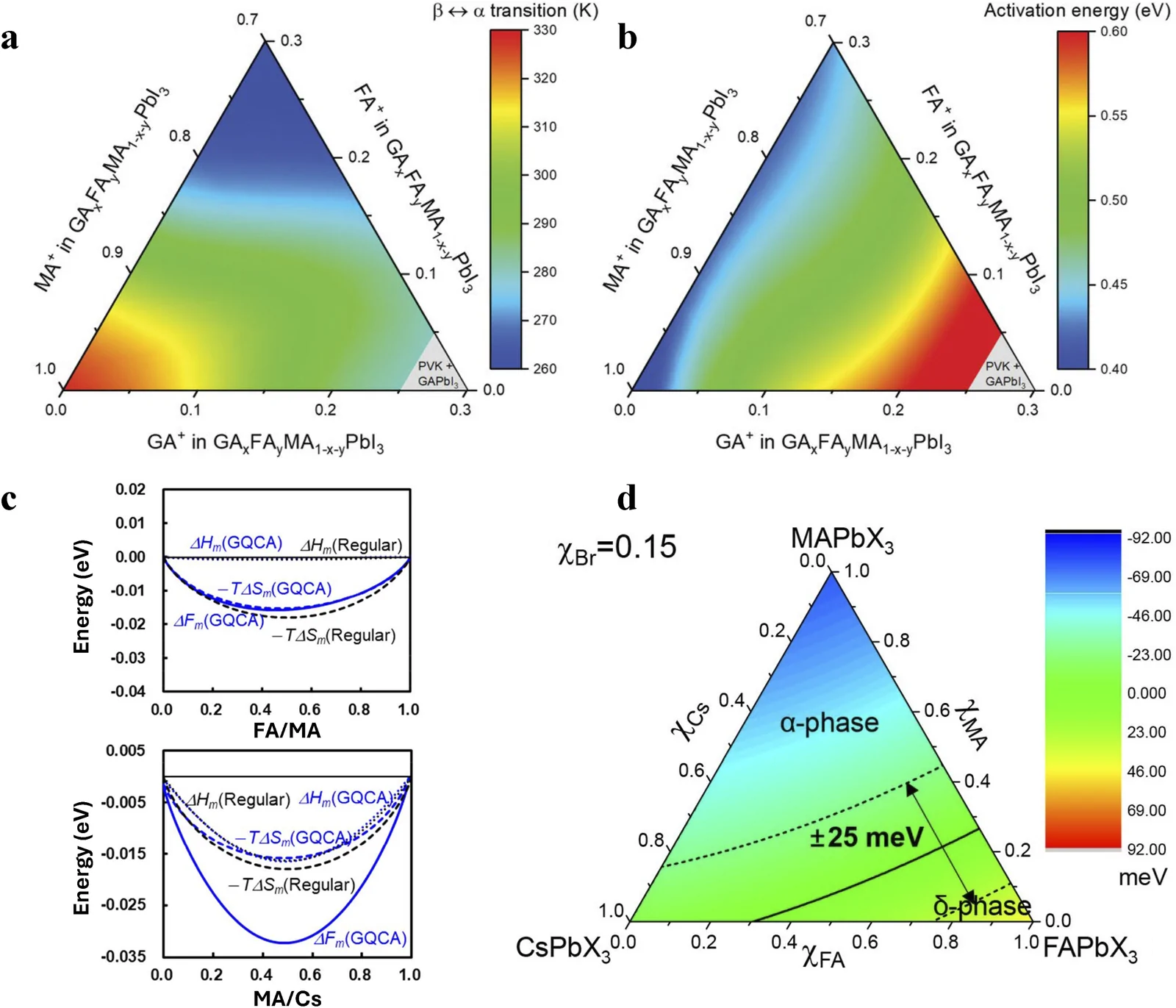
Entropy-driven phase stabilization and transport modulation in mixed A-site perovskite halides. (a) Composition dependent tetragonal-to-cubic phase transition temperatures in the GA_*x*_FA_*y*_MA_1−*x*−*y*_PbI_3_ system, showing enhanced stabilization of the cubic phase toward FA-rich compositions. (b) Composition-dependent ionic transport activation energies in GA_*x*_FA_*y*_MA_1−*x*−*y*_PbI_3_, showing increased ionic-transport activation energies in mixed-cation compositions, especially toward GA-rich regions. Reproduced with permission.^60^ Copyright 2024, Wiley-VCH GmbH. (c) Free energy of mixing for FA–MA alloying and MA–Cs alloying, showing entropy dominated stabilization of mixed-cation perovskites. (d) Cooperative α-phase stabilization of FAPbI_3_ through multicomponent alloying with MA, Cs, and Br at *χ*_Br_ = 0.15. Reproduced with permission.^61^ Copyright 2020, American Chemical Society.

In the GA_*x*_FA_*y*_MA_1−*x*−*y*_PbI_3_ system, Minussi *et al.* systematically mapped how mixed A-site organic cations regulate phase transitions, dielectric response, and ionic transport across the MA, FA, and GA compositional space.^60^ The tetragonal-to-cubic phase transition temperature progressively decreases as MA^+^ is replaced by FA^+^ and GA^+^ cations, indicating stabilization of the high-symmetry cubic phase in mixed-cation perovskites (Fig. 3a). Notably, FA-rich compositions lower this transition temperature more effectively than GA-rich compositions, even though GA^+^ is larger than FA^+^, showing that cubic-phase stabilization cannot be explained by cation size alone. Instead, the difference arises from coupled effects of orientational dynamics and hydrogen bonding: FA^+^ contributes more strongly to orientational entropy because of its faster rotational motion, whereas GA^+^ has more N–H bonds and stronger interactions with the PbI_6_ framework, which restrict rotational freedom. The same composition-property mapping shows that ionic conductivity decreases with increasing substitution content, which was attributed to reduced point-defect density arising from entropy-of-mixing stabilization. In addition, the extracted activation energies are higher in mixed compositions than in pure MAPbI_3_ and increase toward GA-rich regions, reaching values close to 0.6 eV in the composition map (Fig. 3b). These results show that A-site compositional complexity can stabilize high-symmetry phases and suppress iodide-related ion transport, but the outcome depends on the specific balance among cation size, hydrogen bonding, and rotational freedom rather than on configurational entropy alone.

The thermodynamic basis of multicomponent stabilization becomes clearer when FA–MA–Cs mixing behaviour is considered. Kim *et al.* investigated A-site mixing thermodynamics using *ab initio* calculations and showed that the absolute mixing enthalpies remained below 20 meV per formula unit for the FA–Cs, FA–MA, and MA–Cs binary mixtures, indicating that the enthalpic contribution is relatively small and that configurational entropy plays a major role in cation mixing.^61^ In particular, the mixing enthalpy of FA–MA in the α-phase remained close to zero, and its negative mixing free energy was therefore dominated by the entropy contribution, resulting in near-ideal solution behaviour (Fig. 3c). However, MA–Cs mixing also benefited from favourable enthalpic interactions, whereas FA–Cs mixing was comparatively less favourable. Moreover, MA was more effective than Cs in stabilizing the photoactive α-phase relative to the δ-phase, while Cs primarily improved chemical stability against decomposition. These results indicate that multicomponent stabilization arises from configurational entropy together with pair-specific enthalpic interactions and the distinct roles of individual A-site cations.

The stabilizing role of configurational entropy becomes even more pronounced when both A- and X-site alloying are considered simultaneously (Fig. 3d). The calculations further revealed that MA mixing minimizes the Br content required for α-phase stabilization, whereas Cs contributes primarily to chemical stabilization against decomposition rather than direct α-phase stabilization. The thermodynamic analysis further clarifies this division of roles. Cs improves chemical stability against precursor formation and increases the FA-I Schottky defect formation energy from 0.866 eV in FAPbI_3_ to approximately 0.96–0.98 eV, whereas MA contributes more directly to α-phase stabilization. These results suggest that high configurational complexity in multicomponent perovskites should be understood not simply as compositional disorder, but as a cooperative thermodynamic stabilization strategy in which distinct cation and halide components play complementary roles in regulating phase stability, chemical robustness, and ionic transport behaviour.

Beyond thermodynamic stabilization, entropy-driven disorder can also suppress phase inhomogeneity and defect-mediated degradation pathways. Tian *et al.* showed that increasing the number and alkyl-chain flexibility of mixed alkylammonium A-site cations in layered A_2_PbI_4_ hybrid perovskites stabilizes a fully mixed single-phase structure instead of compositionally segregated layered domains because the larger configurational entropy of the organic sublattice counteracts the tendency toward phase separation.^4^ Specifically, equimolar mixing of only two cations, 4C and 12C, produced mixed phases, whereas equimolar mixing of five cations, 4C, 6C, 8C, 10C, and 12C, yielded a single-phase high-entropy perovskite halide. Single-crystal analysis revealed that this disorder is accommodated primarily within the organic interlayers while the inorganic PbI_6_ framework remains ordered, indicating that entropy can improve structural and thermal robustness without directly perturbing the band-edge electronic structure. Consistent with this interpretation, transient absorption revealed a single spectral feature, *in situ* X-ray diffraction (XRD) showed delayed PbI_2_ formation during heating at 150 °C, and molecular dynamics simulations showed that the entropy contribution from flexible alkyl chains increases from 4.24 × 10^−19^ J K^−1^ for 4C to 7.12 × 10^−19^ J K^−1^ for 12C.

Entropy-enhanced orientational disorder has also been linked to suppression of defect-mediated degradation in FA-based perovskites. Chen *et al.* demonstrated that incorporation of 2-amino-1,3,4-thiadiazole (2NTD) into FA-based photoactive perovskites induces a higher-entropy FA^+^ configuration while retaining favourable interactions with the surrounding PbI_6_ octahedral framework.^62^ Polarized Raman measurements further showed reduced orientational preference of FA^+^ after 2NTD modification, supporting the formation of a more dynamically disordered FA^+^ environment. Under accelerated aging at 50 °C in air, the control sample exhibited a distinct I_3_^−^ absorption peak near 360 nm within 3 h, whereas no detectable I_3_^−^ signal appeared in the 2NTD-modified system even after 48 h. Because I_2_/I_3_^−^ accumulation correlates strongly with lattice distortion, vacancy generation, and trap-state formation, these findings indicate that entropy enhanced FA^+^ configurations can improve optoelectronic stability primarily by suppressing defect-mediated degradation pathways rather than by directly altering the band-edge electronic structure.

Taken together, A-site entropy engineering is best understood as a route to lattice-coupled optoelectronic modulation. Its primary consequences include stabilization of high-symmetry phases, suppression of ion migration, regulation of transport behaviour, reduced phase inhomogeneity, and enhanced thermodynamic and environmental robustness. Rather than acting through direct reconstruction of the electronic band structure, configurational entropy primarily regulates the dynamic and thermodynamic landscape of perovskite halides through cooperative disorder, orientational dynamics, and multicomponent mixing effects.

### 3.2 B-site entropy engineering and band-edge optoelectronic modulation

B-site entropy engineering directly modifies the electronic structure of perovskite halides because the band-edge states are governed primarily by metal-halide orbital interactions within the inorganic octahedral framework. Unlike A-site entropy engineering, which mainly affects lattice dynamics and ionic transport, B-site multicomponent alloying reconstructs orbital hybridization, local symmetry, and electronic-state distribution, thereby fundamentally altering optical transitions and excitonic behaviour.

**Fig. 4:**
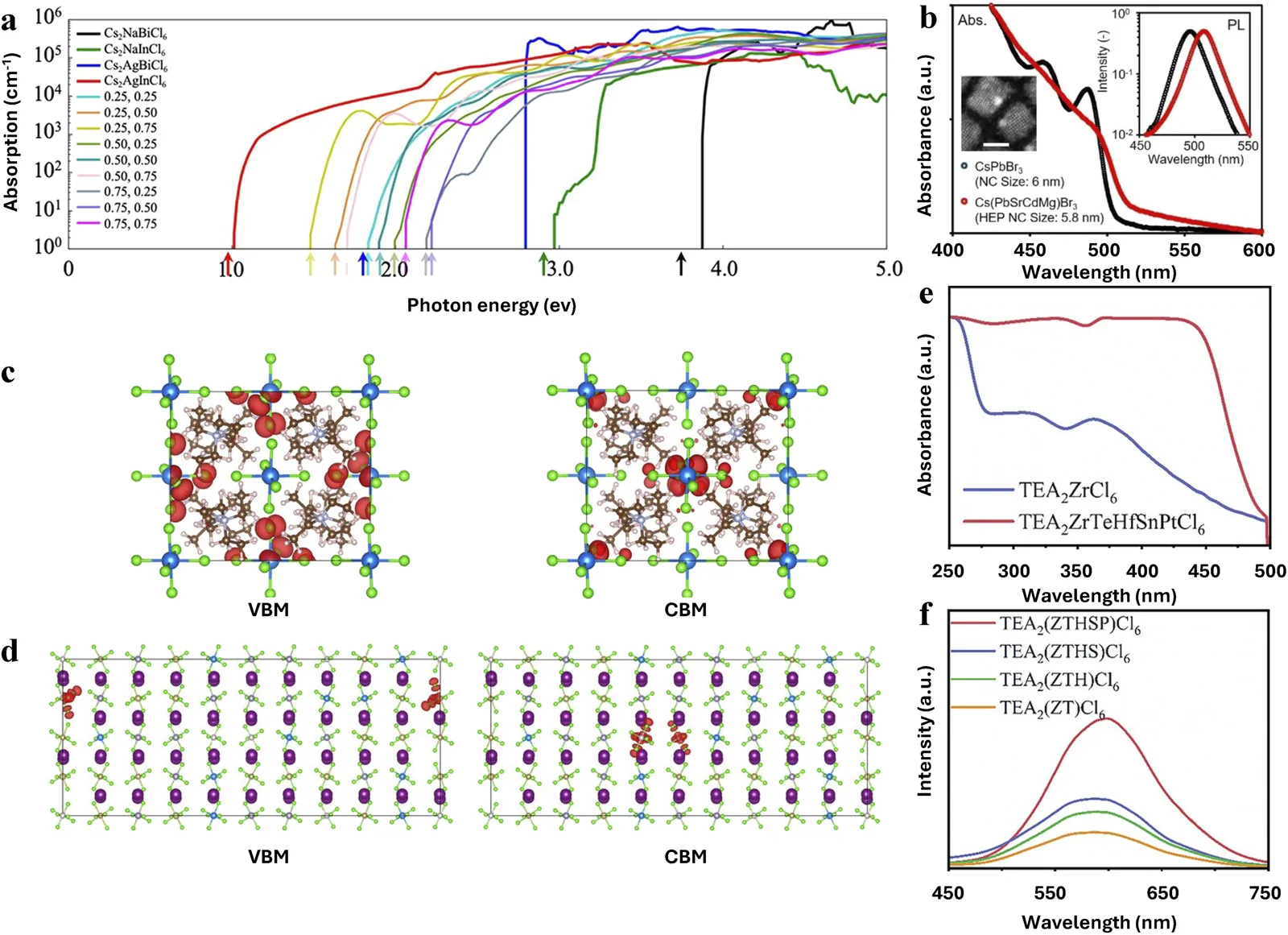
Disorder induced electronic structure reconstruction in high-entropy perovskite halides. (a) Expanded and continuously tunable optical absorption in multielement halide double perovskite alloys arising from entropy stabilized B-site mixing and relaxation of parity forbidden transitions. Reproduced with permission.^15^ Copyright 2022, American Chemical Society. (b) Suppression of excitonic absorption features and emergence of low-energy absorption tails in high-entropy perovskite nanocrystals, indicating disorder induced shallow-state formation and reconstruction of excitonic behaviour. Reproduced with permission.^59^ Copyright 2025, Wiley-VCH GmbH. (c and d) Spatial redistribution of the valence-band maximum and conduction-band minimum in high-entropy hybrid perovskites induced by multicomponent orbital hybridization and electronic-state localization. (e) Broadening of optical absorption following multicomponent alloying. (f) Emergence of intense golden emission associated with disorder induced localized emissive states. Reproduced with permission.^56^ Copyright 2026, Wiley-VCH GmbH.

One important consequence of configurational disorder is entropy-driven stabilization of multicomponent electronic structures. Wang *et al.* showed that multielement alloying in halide double perovskites dramatically expands the accessible absorption range compared to single-component systems, producing continuously tunable absorption onsets across a broad energy window.^15^ This behaviour originates from configurationally stabilized mixing between multiple B-site cations, which modifies the orbital contributions near the band edges and relaxes symmetry restricted electronic transitions (Fig. 4a). In particular, alloy induced local symmetry breaking partially relaxes parity forbidden transitions that normally suppress optical absorption in ordered double perovskites, thereby enhancing band-edge absorption and enabling broader electronic tunability.

Configurational disorder also fundamentally reconstructs excitonic behaviour by generating shallow localized electronic states near the band edge. Chiang *et al.* demonstrated that high-entropy perovskite NCs exhibit substantially weakened excitonic absorption features and pronounced low-energy absorption tails compared to conventional CsPbBr_3_ NCs, even though both systems possess similar particle sizes below the Bohr exciton diameter.^59^ For example, Cs(PbSrCdMg)Br_3_ high-entropy NCs with an average size of 5.7 nm showed no discernible confined excitonic resonance, whereas 6 nm CsPbBr_3_ NCs displayed a clear absorption peak at 475 nm associated with quantized density of states. The spectra show that the excitonic absorption peak is strongly suppressed in the high-entropy system, while the absorption tail progressively extends toward lower energies (Fig. 4b). These observations indicate that conventional quantum confinement behaviour becomes disrupted in high-entropy systems because compositional disorder reduces band dispersion and generates shallow near-band-edge states. Rather than forming well-defined excitonic states, carriers experience spatially fluctuating electronic environments arising from multi-component B-site disorder, leading to reconstruction of the band-edge electronic structure. Time-resolved photoluminescence supports the shallow-state-mediated recombination picture. Upon high-entropy alloying, the average photoluminescence lifetime decreases from 48.4 ns in parent CsPbBr_3_ NCs to as low as 6.1 ns, while the exciton binding energy increases from about 70 meV in CsPbBr_3_ to up to 180 meV in trinary alloyed NCs. The shallow near-band-edge states therefore do not behave as deep non-radiative traps, but instead promote rapid radiative recombination through enhanced carrier localization near the band edge.

The electronic origin of this behaviour becomes clearer when the spatial distribution of the band-edge states is considered. Liu *et al.* showed that multicomponent alloying in high-entropy hybrid perovskites reconstructs the orbital character of the electronic states through disorder-induced electronic redistribution.^56^ In addition, the valence-band maximum and conduction-band minimum become spatially redistributed because the incorporation of multiple heavy metal cations modifies the orbital contributions within the inorganic framework (Fig. 4c and d). Incorporation of Pt and Te reconstructs the conduction-band electronic density and introduces localized electronic regions associated with the metal-halide network. This disorder induced orbital redistribution promotes electronic-state localization and reduces the spatial overlap associated with conventional delocalized band-edge states. Liu *et al.* further demonstrated that this electronic-state redistribution directly modifies the optical response of the material.^56^ Multicomponent alloying significantly broadens optical absorption relative to the parent TEA_2_ZrCl_6_ system, indicating formation of multiple electronically accessible states across the band structure (Fig. 4e). Simultaneously, increasing configurational complexity progressively enhances and broadens the emission response, producing intense golden emission centred near 590 nm (Fig. 4f). Rather than arising from a simple superposition of individual component emissions, this behaviour originates from disorder induced localized emissive states formed through multicomponent orbital hybridization and electronic-state redistribution. Together, these results demonstrate that B-site entropy engineering not only reconstructs the band-edge electronic structure, but also generates new radiative recombination pathways that are inaccessible in compositionally ordered systems.

Beyond band-edge reconstruction, B-site entropy engineering can further reshape excited-state relaxation pathways. Whereas undoped CsPbCl_3_ quantum dots show only host exciton emission around 415 nm, Zhao *et al.* reported that Cs(Pb_0.2_Mn_0.2_Ni_0.2_Zn_0.2_Yb_0.2_)Cl_3_ quantum dots exhibit additional Mn^2+^ emission around 600 nm and Yb^3+^ near-infrared emission around 980 nm, indicating that band-edge excitation is no longer confined to the host but is coupled to dopant-centred emission channels.^63^ This change is accompanied by a blue shift of the absorption edge from 436 to 429 nm, corresponding to a bandgap widening from 2.90 to 2.94 eV, showing that high-entropy B-site alloying also modifies the host electronic structure. The shortened average fluorescence lifetime of 8.36 ns further supports more efficient host-to-dopant energy transfer in a defect-passivated lattice. Ding *et al.* further showed that B-site disorder can extend band-edge reconstruction into the regime of quantum emission.^64^ In high-entropy CsPb(Br/Cl)_3_ perovskite NCs alloyed with multiple divalent B-site cations, Mn^2+^, Zn^2+^, and Ni^2+^, compositional disorder promoted exciton localization, reduced electron–phonon coupling, and suppressed trap-assisted recombination, yielding deep-blue room-temperature single-photon emission with 96% purity and *g*^2^(0) = 0.04, indicative of strong antibunching and nearly ideal single-photon emission. Together, these results show that B-site disorder can reshape the local excited-state landscape from host-dominated relaxation toward coupled and spatially confined radiative channels.

The effects of B-site entropy engineering are not monotonic. In unequal high-entropy Cs(Mn_*x*_Ni_1−*x*/4_Ca_1−*x*/4_Mg_1−*x*/4_Pb_1−*x*/4_)Cl_3_ quantum dots, Zhu *et al.* showed that the Mn^2+^-related visible emission depends non-monotonically on Mn content.^65^ The emission around 600 nm reached its maximum at 30% Mn, where it was enhanced by 200% relative to the equimolar high-entropy composition Cs(Mn_0.2_Ni_0.2_Ca_0.2_Mg_0.2_Pb_0.2_)Cl_3_ and still retained about 85% after 50 days. When the Mn content exceeded 30%, however, the bandgap decreased to 2.87 eV, pinned defect states broadened the conduction band, and the visible emission dropped sharply. A similar trend appears in Pb-containing ternary and higher-order B-site alloyed CsPbBr_3_ derived NCs, where moderate alloying enhances exciton binding and radiative recombination, but excessive alloying weakens exciton binding and reduces photostability.^59^ These results show that the beneficial role of B-site entropy is restricted to a composition window beyond which disorder becomes defect-generating and degrades photophysical quality.

Overall, B-site entropy engineering is best viewed as a powerful means of reconstructing the band-edge electronic landscape. Its characteristic signatures include tuneable bandgaps, modified carrier effective masses, relaxed optical selection rules, shallow-state formation, altered exciton binding, and energy-transfer-assisted luminescence. However, these advantages are realized only within an optimum composition window, because excessive B-site heterogeneity can broaden the band edge unfavourably, generate defect states, and undermine photophysical stability.

### 3.3 X-site entropy engineering and mixed-halide thermodynamics

X-site compositional engineering directly modifies the metal-halide framework and therefore provides strong control over bandgap, emission energy, and halide-ion dynamics. However, mixed-halide systems should not automatically be classified as high-entropy materials. Most available studies concern binary I/Br or Br/Cl alloys and are more appropriately used to establish the thermodynamic and kinetic principles that govern X-site mixing. Direct experimental evidence for high-entropy X-site design is currently provided by triple-halide Cl/Br/I compositions stabilized through simultaneous regulation of the metal sublattice.^23^

**Fig. 5:**
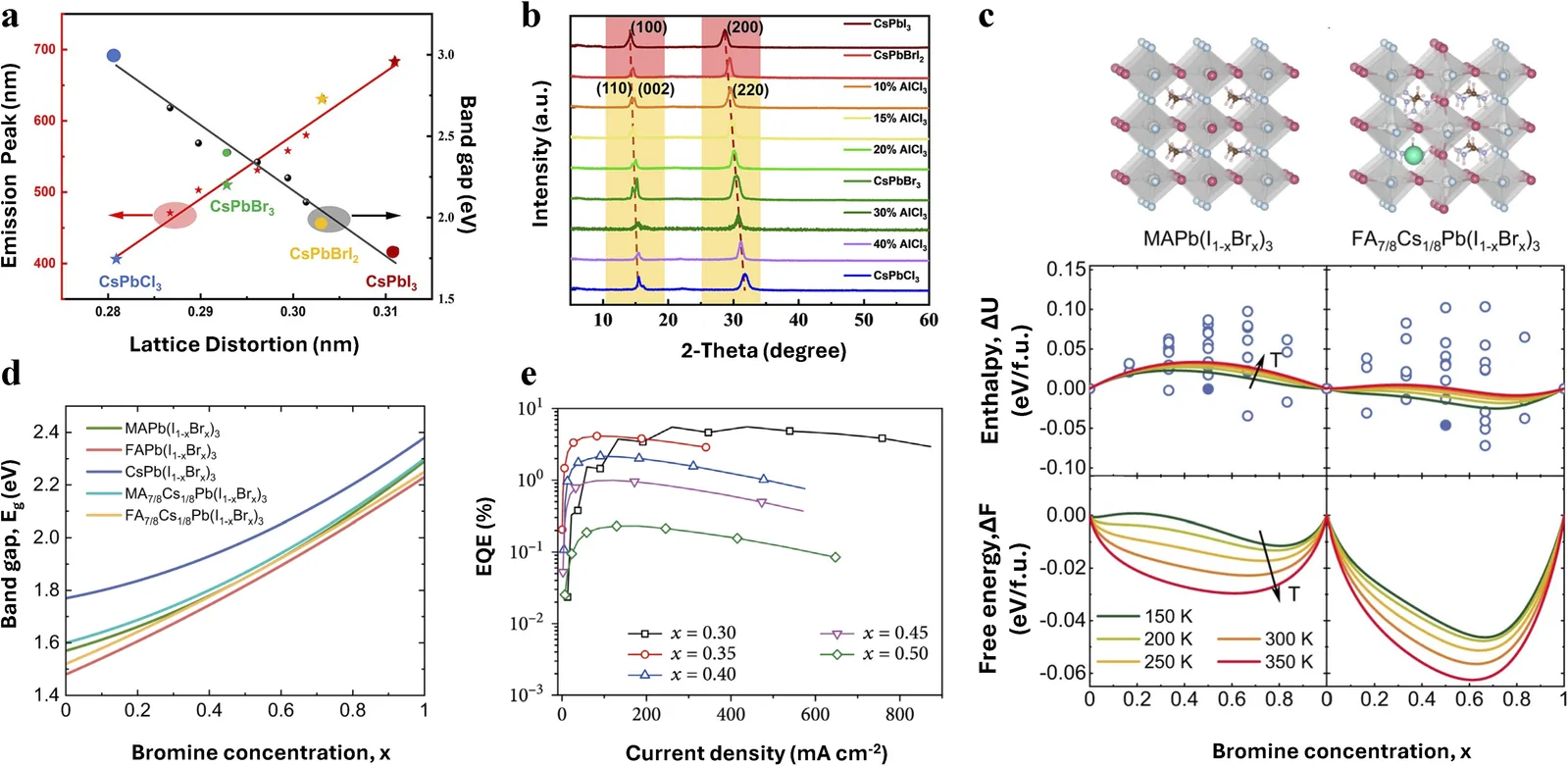
Structural, thermodynamic, and device-level characteristics of X-site-engineered mixed-halide perovskites. (a) Linear dependence of emission peak and bandgap on lattice distance in CsPb_1−*a*_Al_*a*_(Cl_1−*x*−*y*_Br_*x*_I_*y*_)_3_ NCs. (b) XRD patterns of CsPb_1−*a*_Al_*a*_(Cl_1−*x*−*y*_Br_*x*_I_*y*_)_3_ NCs. Reproduced with permission.^23^ Copyright 2025, American Chemical Society. (c) Representative atomic structures of the most stable mixed-halide configurations at a relative Br concentration of *x* = 0.5, together with the corresponding mixing enthalpy Δ*U*, and mixing free energy Δ*F*. (d) Band gap as a function of relative Br concentration *x* for the different compounds. Reproduced with permission.^66^ Copyright 2021, Springer Nature. (e) Characterization of perovskite LEDs based on CsPb(Br_1−*x*_Cl_*x*_)_3_ films. EQE *versus* current density. Reproduced with permission.^69^ Copyright 2020, Science and Technology Review Publishing House.

A direct route to synthesizing triple-halide high-entropy perovskites was demonstrated through octahedral-factor regulation. Post-treatment of CsPbBrI_2_ nanocrystals with AlCl_3_ introduced both Al^3+^ and Cl^−^, reducing the octahedral-factor difference between the Cl-, Br-, and I-containing coordination environments. At an Al/Pb ratio of 18%, the calculated octahedral-factor difference between the corresponding Cl- and I-based octahedra decreased from 0.116 to 0.107. The authors associated this change, together with shorter metal-halide bonds and stronger Al-X bonding, with suppression of crystal distortion and halide migration. Under these optimized conditions, octahedral-factor regulation enabled the formation of a single-phase triple-halide perovskite with the composition Cs_1.06_Pb_0.85_Al_0.15_(Cl_0.29_Br_0.46_I_0.25_)_2.43_. The same regulation concept was also extended to Zn^2+^, Mn^2+^, and In^3+^. The emission peak and bandgap exhibited an approximately linear dependence on lattice distance (Fig. 5a), while the corresponding XRD patterns showed systematic structural evolution across the metal-chloride-treated triple-halide nanocrystals (Fig. 5b).^23^

The central challenge is the competition between mixing entropy and the energetic penalties associated with dissimilar halide environments. Mixed-halide perovskites can be thermodynamically stable in the dark yet separate under illumination into domains with different compositions and bandgaps. The thermodynamic model developed for I/Br perovskites attributes this behaviour to the lowering of photocarrier free energy when carriers funnel into nucleated, lower-bandgap I-rich regions. The predicted threshold photocarrier density is governed primarily by the bandgap difference between the parent alloy and the I-rich phase, while removal of illumination allows the homogeneous mixed state to recover. Calculated atomic configurations, mixing enthalpies (Δ*U*), and mixing free energies (Δ*F*) further show that the thermodynamic landscape of X-site mixing varies with Br concentration, temperature, and A-site composition (Fig. 5c).^66,67^

This framework clarifies why configurational entropy alone cannot guarantee operational phase stability. The thermodynamic bandgap model treats the illuminated free energy as the sum of the dark mixing free energy and a photocarrier contribution. Illumination can therefore make phase separation favourable even when entropy stabilizes the mixed composition in the absence of light. The same model predicts excitation-intensity thresholds below which segregation is suppressed and a lower tendency toward segregation in nanocrystals than in extended films. The calculated bandgap also varies systematically with relative Br concentration and A-site identity (Fig. 5d), thereby modifying the bandgap contrast that governs photocarrier funnelling into lower-bandgap I-rich regions. These results indicate that X-site stability depends jointly on mixing thermodynamics, bandgap contrast, photocarrier density, and the length scale over which carriers and halides can redistribute.^66,67^

The local lattice environment provides an additional control parameter. In FAPbBr_1.8_I_1.2_, substitution of 10% MA at the A site increased the measured activation energy for photoinduced segregation from 27.65 ± 1.28 to 32.35 ± 1.28 kJ mol^−1^. This result shows that modification of the surrounding lattice can raise the energetic barrier for halide redistribution without changing the nominal Br/I composition. The broader implication is that X-site behaviour cannot be considered independently of the A- and B-site environments, because both can alter octahedral distortion, bandgap contrast, and the barrier to ion motion.^68^

The importance of compositional homogeneity is also evident in Br/Cl blue-emitting perovskites. Halide heterogeneity was detected in as-deposited films and traced to nonuniform mixing in the precursor solution. Introducing cationic surfactants improved precursor-level halide homogeneity and produced spectrally stable, single-phase blue-emitting films. The corresponding EQE-current-density characteristics of CsPb(Br_1−*x*_Cl_*x*_)_3_ LEDs demonstrate that electroluminescence efficiency and roll-off depend strongly on the Br/Cl composition (Fig. 5e). This result does not itself demonstrate a high-entropy composition, but it shows that macroscopic phase stability and device performance depend on achieving homogeneous halide distribution before and during crystallization.^69^

Taken together, X-site entropy engineering is best viewed as a coupled thermodynamic and structural design problem. Increasing halide diversity can expand configurational entropy and optical tunability, but these benefits are opposed by bandgap-driven photosegregation, octahedral mismatch, and halide migration. The available evidence therefore supports a design strategy in which X-site complexity is combined with regulation of the surrounding lattice, precursor homogeneity, and carrier-induced free-energy changes.

## 4. Synthesis, characterization, and computational design of high-entropy perovskite halides

### 4.1 Synthetic routes for high-entropy perovskite halides

#### 4.1.1 Mechanical ball milling

**Fig. 6:**
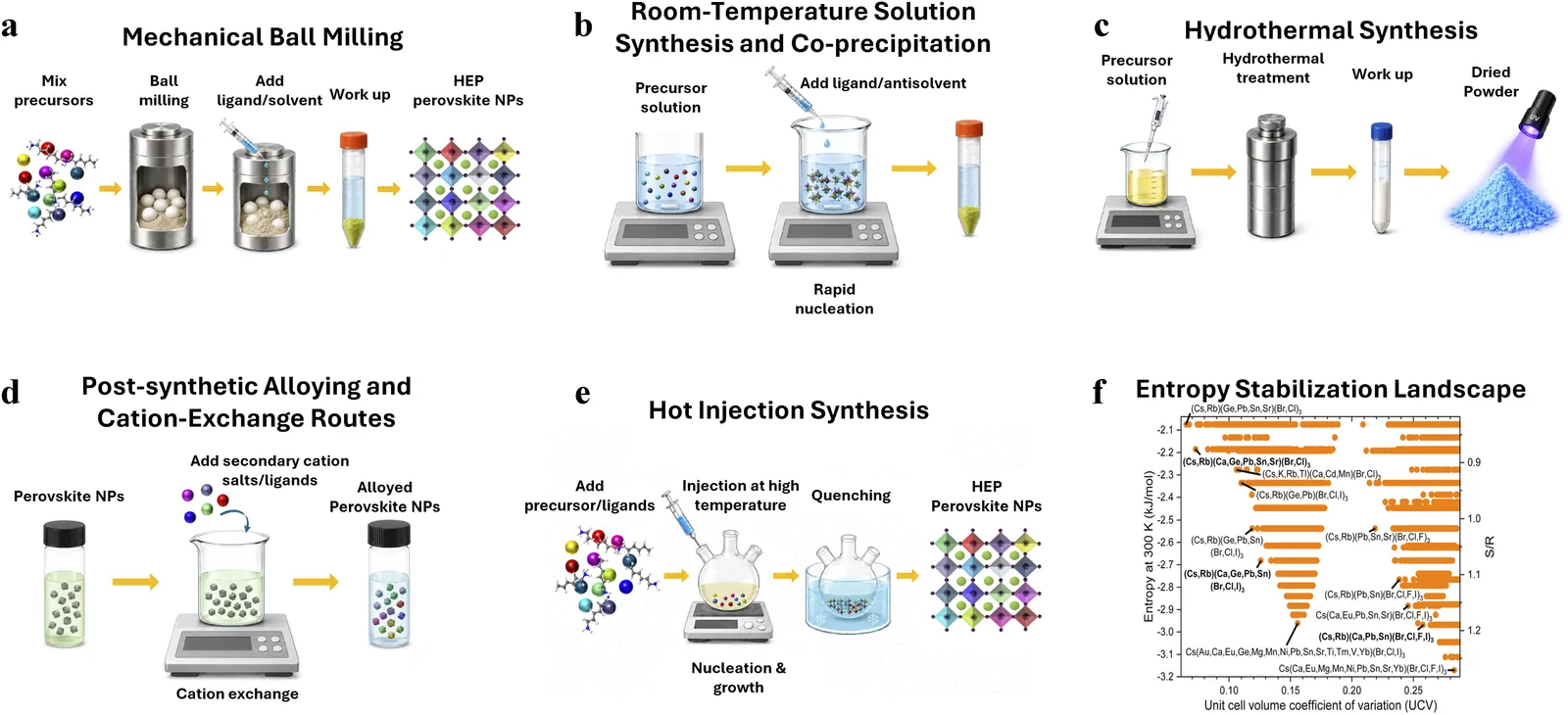
Representative synthesis routes and entropy-stabilization landscape of high-entropy perovskite halides. (a) Mechanical ball milling, (b) room-temperature solution synthesis and co-precipitation, (c) hydrothermal synthesis, (d) post-synthetic alloying and cation-exchange routes, and (e) colloidal nanocrystal synthesis for the preparation of high-entropy perovskite halides. (f) Entropy stabilization (at 300 K) plotted as a function of the enthalpic penalty, represented by the unit-cell volume coefficient of variation (UCV), for all equimolar inorganic high-entropy perovskite halide compositions with experimentally observed constitutive end-members and mixing on all sublattices. Promising alloy compositions are labeled in bold. Reproduced with permission.^20^ Copyright 2024, the Royal Society of Chemistry.

High-energy ball milling is one of the most direct routes for preparing all-inorganic high-entropy perovskite halides, particularly in nanocrystal form. This method relies on repeated fracture, cold welding, and forced mixing of solid precursors, making it especially suitable for multication systems that are difficult to homogenize under equilibrium conditions (Fig. 6a). Zhao *et al.* used ball milling to prepare Cs(Pb_0.2_Mn_0.2_Ni_0.2_Zn_0.2_Yb_0.2_)Cl_3_ quantum dots, showing that five metal cations could be incorporated into a single perovskite lattice through a rapid solid-state process.^63^ A related lead-free example was reported by Qiu *et al.* where Cs(Na_0.2_Bi_0.2_Mn_0.2_Ni_0.2_Zn_0.2_)Cl_3_ NCs were synthesized within 20 minutes.^70^ These studies highlight the main advantages of ball milling, namely its rapid processing, solvent-free nature, and potential scalability. However, strong mechanical forcing can also lead to broader particle-size distributions, reduced control over morphology, and greater difficulty in confirming true local compositional homogeneity. Comparatively, ball milling is most appropriate when rapid, solvent-free incorporation of multiple inorganic cations is prioritized over precise control of particle morphology and local compositional uniformity.

#### 4.1.2 Room-temperature solution synthesis and co-precipitation

A second important route is room-temperature solution synthesis, often combined with co-precipitation, which takes advantage of the soft ionic nature of perovskite halides and their ability to crystallize under very mild conditions (Fig. 6b). Zhang *et al.* introduced a rapid room-temperature route under air for entropy-stabilized colloidal NCs, and successfully synthesized Cs(PbSnSrMnZn)Br_3_ with peak emission at 525 nm, emphasizing that such a mild process contrasts sharply with the high-temperature routes typically required for high-entropy perovskites.^18^ Likewise, Liu *et al.* reported that the organic–inorganic hybrid high-entropy perovskite (TEA)_2_(Zr_0.18_Te_0.22_Hf_0.2_Sn_0.3_Pt_0.1_)Cl_6_ could be formed immediately by co-precipitation at 20 °C after mixing hydrochloric acid solutions containing tetraethylammonium chloride and multiple tetravalent metal ions.^56^ The main advantages of this route are its mild reaction conditions, low energy input, and direct access to entropy-stabilized products during nucleation. However, because crystallization occurs rapidly, it can also be more difficult to fully control particle size, phase purity, and local compositional uniformity. Compared with mechanochemical processing, this route offers milder and more direct solution-phase crystallization but provides less control when nucleation and multicomponent incorporation occur simultaneously.

#### 4.1.3 Hydrothermal synthesis

Hydrothermal synthesis has been used mainly for high-entropy double perovskites and related chloride crystals, where a closed solution environment supports slow crystal growth and dopant incorporation (Fig. 6c). Xue *et al.* prepared single crystals with general formula Cs_2_M^I^M^III^Cl_6_ through a hydrothermal route, mainly introducing entropy at the M^III^ (In^3+^, Sb^3+^, Bi^3+^, Tb^3+^, Dy^3+^, Er^3+^, Ho^3+^, Yb^3+^) site while maintaining crystallinity and phase purity.^31^ Its relevance is also supported by earlier work on non-high-entropy double-perovskite by Li *et al.*, where rare-earth-doped Cs_2_(Na, Ag)InCl_6_ was synthesized hydrothermally.^71^ Although that study is not a high-entropy example, it illustrates why hydrothermal methods are well suited to halide double-perovskite crystals intended for optical applications. The main advantages of hydrothermal synthesis are its ability to produce highly crystalline products, support dopant incorporation, and maintain phase purity in a chemically controlled growth environment. However, it is generally slower than room-temperature precipitation or mechanochemical routes and is less convenient for rapid large-scale production of NCs. Accordingly, hydrothermal synthesis is preferable when crystallinity, phase purity, and dopant accommodation are more important than rapid processing or nanocrystal-scale production.

#### 4.1.4 Postsynthetic alloying and cation-exchange routes

For colloidal NCs, postsynthetic alloying and cation-exchange-type routes are especially useful because they allow compositional complexity to be introduced while preserving the original nanocrystal morphology (Fig. 6d). Solari *et al.* used a post-synthetic strategy based on ligand-assisted solid-phase synthesis, starting from colloidal MAPbBr_3_ NCs and then introducing Mg^2+^, Sn^2+^, Cd^2+^, and Zn^2+^ as secondary cations into the B-site.^72^ A related approach was reported by Chiang *et al.* research, where CsPbBr_3_ NCs were first synthesized by ligand-assisted reprecipitation and then subjected to ligand-assisted multicationic exchange using excess divalent metal bromide salts.^59^ These postsynthetic routes are particularly important in nanocrystal systems because direct one-step synthesis may not always provide sufficient control over both particle morphology and multication incorporation. Their main advantage is that alloying can be introduced after nanocrystal formation, which helps preserve morphology while enabling stepwise compositional tuning. However, cation exchange may remain incomplete or nonuniform, making it more difficult to confirm whether the final NCs are compositionally homogeneous throughout the entire lattice. Relative to direct multicomponent synthesis, postsynthetic exchange provides better retention of nanocrystal morphology, although this advantage is offset by a greater risk of incomplete or spatially nonuniform alloying.

#### 4.1.5 Hot injection synthesis

A more specialized route is hot injection synthesis designed for strongly confined quantum dots, especially when spectral purity and device stability are critical (Fig. 6e). Guo *et al.* prepared entropy-driven pure-red ABI_3_ quantum dots with simultaneous alloying at the A- and B-sites, producing GA_0.28_ Cs_0.72_Pb_0.40_Ge_0.55_Cd_0.05_I_3_ NCs.^73^ Notably, these NCs exhibited pure red emission at 632 nm with an almost near-unity PLQY of ≈100%, demonstrating the effectiveness of entropy-driven compositional engineering in achieving highly efficient red emission. This example shows that, for highly confined emitters, the synthesis route must control not only multicomponent incorporation but also the nanocrystal size and confinement regime, as these directly affect spectral stability and emission behavior. The main advantage of this route is the high degree of control it offers over nanocrystal size, optical properties, and emission colour. However, it also demands stricter control over precursor chemistry, reaction conditions, and surface ligands, which can make reproducibility and scale-up more difficult. Thus, hot injection is most suitable for optically demanding nanocrystal applications requiring tight control over size and emission, whereas its synthetic complexity makes it less attractive for simple or large-scale production.

#### 4.1.6 General remarks on route selection

Overall, the synthetic routes used for high-entropy perovskite halides are chosen according to the form of the final material and the difficulty of achieving homogeneous multicomponent incorporation. Ball milling is most effective for rapidly forcing multication mixing in inorganic powders and NCs. Room-temperature solution processing and co-precipitation are particularly suitable for soft ionic halide systems that can crystallize directly into entropy-stabilized phases. Hydrothermal synthesis is better suited to bulk chloride crystals and high-entropy double perovskites, while postsynthetic alloying and cation exchange are especially useful for colloidal NCs whose morphology must be retained during compositional modification. Across all these methods, the central synthetic goal is the same: to introduce multiple principal components in a way that produces a structurally coherent and compositionally homogeneous halide phase rather than a nominally mixed but microscopically segregated product.

### 4.2 Experimental verification of high-entropy characteristics

Assigning high-entropy character requires more than confirming the presence of multiple elements. Experimental verification should establish both the actual configurational-entropy regime and the degree of compositional coherence across relevant length scales. The measured elemental ratios should support the stated entropy classification, and the constituent species should occupy the intended crystallographic sublattice within a coherent mixed phase rather than forming a physical mixture of isostructural phases or compositionally distinct nanodomains. Powder X-ray diffraction (PXRD) is essential for determining the average crystal structure and detecting sufficiently abundant secondary phases, but it cannot independently distinguish homogeneous substitutional disorder from nanoscale segregation when the constituent phases have similar structures and lattice parameters. Peak broadening may arise from compositional disorder, crystallite size, strain, or the superposition of multiple phases. Energy-Dispersive X-ray Spectroscopy (EDX) mapping provides complementary information on micrometre- or nanometre-scale elemental distributions but generally cannot verify atomic-level mixing or exclude persistent nanodomains, particularly when the concentration of each element approaches its detection limit. Single-crystal diffraction does not necessarily remove this ambiguity because different mixed-occupancy models may yield similarly acceptable refinements, and an individual crystal may not represent a heterogeneous polycrystalline bulk sample. Phan *et al.* demonstrated these limitations directly in multielement Cs_2_BCl_6_ systems for which PXRD and EDX appeared consistent with homogeneous phase formation even when local phase separation was present.^22^

Solid-state nuclear magnetic resonance (NMR) has provided particularly direct information on atomic-scale elemental distributions in Cs_2_BCl_6_-type materials. Each Cs atoms are surrounded by four neighbouring [BCl_6_]^2−^ octahedra, making ^133^Cs an effective spectator nucleus for probing the identities and arrangements of nearby B-site species. Phan *et al.* compared Cs_2_BCl_6_ compositions containing up to eight B-site elements prepared by solution, hydrothermal, and mechanochemical routes.^22^ Although PXRD and EDX initially suggested successful high-entropy phase formation across the different methods, ^133^Cs NMR revealed multiple non-Gaussian resonances for the solution- and hydrothermally prepared samples, indicating phase-separated or compositionally clustered domains. In contrast, the mechanochemically prepared samples exhibited broad, nearly Gaussian resonances centred near the composition-weighted chemical shifts of the parent compounds. The absence of dominant parent-like resonances and agreement with statistical occupancy models supported substantially more homogeneous B-site mixing. For mechanochemically prepared Cs_2_SnZrCl_6_, the populations of the five possible CsZr_*n*_Sn_4−*n*_ environments agreed with the binomial distribution expected for random occupation of the four neighbouring B sites, whereas hydrothermally prepared material deviated from this distribution and showed preferential clustering. Medium-entropy three- and four-component samples required additional milling to eliminate asymmetric spectral features. By comparison, five to eight component samples produced broad Gaussian-like distributions after shorter milling, which the authors interpreted as evidence that increased configurational entropy facilitated homogeneous incorporation. These results show that local spectra should be evaluated against parent compounds, physical mixtures, and statistically generated occupancy models rather than interpreted from spectral broadening alone.

Solid-state NMR is also valuable for less compositionally complex systems, although its limitations must be considered. Tai *et al.* used ^133^Cs NMR to distinguish local environments in Cs_2_[SnZr]Cl_6_, Cs_2_[SnHf]Cl_6_, Cs_2_ [ZrHf]Cl_6_, and Cs_2_[SnZrHf]Cl_6_ that were not clearly resolved by PXRD or Raman spectroscopy. However, the nearly identical ^133^Cs chemical shifts of Cs_2_ZrCl_6_ and Cs_2_HfCl_6_ show that chemically similar species may produce overlapping resonances.^32^ Low natural abundance, weak sensitivity, paramagnetic broadening, spectral overlap, and the reduced concentration of each element in highly multicomponent systems may further complicate NMR measurements, necessitating multinuclear analysis, isotope enrichment, higher magnetic fields, or computational chemical-shift calculations. Nuclear quadruple resonance (NQR) spectroscopy provides a complementary approach for quadrupolar halide nuclei because its resonance frequencies are highly sensitive to the local electric-field gradient and bonding geometry.^74^ Aebli *et al.* used ^127^I Nuclear Quadrupole Resonance (NQR) to distinguish iodide environments in mixed-cation and mixed-halide FAPbI_3_-derived perovskites that retained an average cubic structure by XRD.^75^ Cs clustering was detected at 10% Cs substitution before macroscopic phase separation became readily detectable by diffraction, whereas low Br substitution produced local environments consistent with homogeneous solid-solution formation. Although these compositions are not formal high-entropy systems, they show that NMR and NQR can identify clustering and local heterogeneity before secondary phases become visible by diffraction.

Total-scattering pair distribution function (PDF) analysis provides complementary real-space information over short and intermediate length scales. PDF analysis can reveal local symmetry lowering, bond-length distributions, octahedral distortions, and short-range structural correlations hidden within a high-symmetry average structure. Studies of conventional CsPbX_3_ perovskites have shown that local orthorhombic distortions may persist even when the average structure appears cubic, with orthorhombic models providing better fits to short-range PDF data.^76^ When applied to high-entropy perovskite halides, fitting over different real-space ranges could help determine whether the measured structure is better described by a single locally distorted mixed phase or by a combination of end-member-like nanodomains.

X-ray absorption near-edge spectroscopy (XANES) and extended X-ray absorption fine structure (EXAFS) provide element-specific information on the electronic and local coordination environments of selected constituent elements. XANES can establish oxidation states and changes in local electronic symmetry, while EXAFS provides coordination numbers, nearest-neighbour bond lengths, and bond-length disorder around the absorbing element. Pradeep *et al.* combined XRD, XANES, EXAFS, and Raman spectroscopy to investigate conventional mixed-halide CsPb(Cl/Br)_3_ nanocrystals across several length scales.^76^ EXAFS distinguished separate Pb–Cl and Pb–Br scattering paths rather than the single compositionally averaged bond length inferred from diffraction and quantified coordination numbers and local disorder around Pb. Together with XRD and Raman spectroscopy, the measurements supported the formation of domain-free mixed-halide solid solutions containing randomly distributed halides around the Pb centres. Although this system is not formally high entropy, it provides a methodological precedent for distinguishing a coherent solid solution from a phase-separated material. EXAFS nevertheless remains primarily sensitive to the nearest-neighbour environment and cannot independently determine whether locally similar octahedra are randomly dispersed or grouped into medium-range nanodomains. It should therefore be combined with techniques that probe longer spatial distributions when elemental clustering is the central question.

No single characterization method can establish high-entropy character across all relevant length scales. Reliable verification therefore requires agreement among bulk compositional analysis, average crystallography, spatially resolved elemental mapping, and local structural probes. Inductively coupled plasma (ICP)-based analysis or related methods can determine the overall elemental ratios, while diffraction and local spectroscopy are needed to confirm that the constituent elements occupy the intended crystallographic sublattice. Configurational entropy should consequently be estimated from experimentally supported site occupancies rather than from nominal precursor ratios or bulk composition alone. XRD establishes the average crystallographic phase, microscopy and elemental mapping evaluate compositional variations from the micrometre to nanometre scale, and solid-state NMR, NQR, PDF analysis, and XANES/EXAFS distinguish coherent atomic-scale mixing from preferential clustering, persistent nanodomains, and residual end-member phases. Short-range ordering may exist within a compositionally coherent high-entropy phase and should not by itself be interpreted as phase separation. High-entropy character should therefore be assigned only when the calculated configurational entropy is supported by local structural evidence for a coherent mixed phase. The need for multiscale verification is particularly relevant to the compositional-homogeneity challenge discussed in Section 6.2.

### 4.3 Computational guidance for high-entropy perovskite halides

High-entropy perovskite halides are increasingly being explored through density functional theory (DFT) calculation, computational screening, and machine-learning (ML)-guided discovery, because the accessible compositional space is too large for direct trial-and-error optimization alone. Muzzillo *et al.* showed that even when only experimentally observed halide-perovskite end members are considered, the number of possible equimolar alloy combinations becomes extremely large, making low-cost pre-screening essential before detailed calculations or experiments.^20^ Their study further identified configurational entropy and the unit-cell volume coefficient of variation (UCV) as practical descriptors for balancing entropy stabilization against structural mismatch. Ahmadi *et al.* similarly emphasized that substitutions at the A, B, and X sites generate a near-infinite compositional space, while target properties such as stability, bandgap, defect tolerance, and photoluminescence are often optimized in different regions of that space.^52^ Together, these studies show that future high-entropy perovskite halide design will increasingly rely on computational pre-screening before experimental validation.

From a site-selection perspective, the A site appears to be the most accessible entry point for entropy engineering because it can host both inorganic and organic monovalent cations while perturbing the electronically active B–X framework less directly than B- or X-site substitution. Muzzillo *et al.* showed that A-site-only mixing is the most conservative strategy, giving a maximum entropy stabilization of 0.97 kJ mol^−1^ at 300 K for (Cs, In, K, Li, Na, Rb, Tl)CaBr_3_ with UCV = 0.063, while preserving more ordered valence and conduction bands.^20^ When the A site is combined with additional disorder, the accessible entropy increases to 1.68 kJ mol^−1^ for A/B-site mixing and 2.77 kJ mol^−1^ for A/X-site mixing, showing that the A sublattice becomes more effective when paired with either B- or X-site alloying. In practice, Cs^+^ functions mainly as a structural baseline, whereas Rb^+^, K^+^, and in some screened cases Tl^+^ appear as entropy-enhancing partners. Organic A-site mixing broadens this design space further: Minussi and Araújo showed that the penta-organic system GA_*x*_FA_*x*_EA_*x*_AC_*x*_MA_1−4*x*_PbI_3_ can remain single phase up to nearly *x* = 0.08, while retaining bandgaps near 1.5 eV, reducing ionic transport, and improving stability.^21^ Overall, A-site entropy engineering is therefore best viewed as a relatively low-disruption route to improved stability and suppressed ion migration.

The B site is more powerful for property tuning because it directly forms the metal-halide framework and therefore more strongly affects the band-edge electronic structure. Ahmadi *et al.* highlighted Sn- and Ge-based chemistries as major Pb-free directions, while Muzzillo *et al.* showed that B-site alloying can generate relatively large entropy gains without always incurring the same penalty as extensive X-site mixing.^20,52^ In their screening, the largest entropy stabilization for all-sublattice mixing was 3.22 kJ mol^−1^ for CsB(Br, Cl, F, I)_3_ with 10 B-site components, while more favourable UCV-entropy trade-offs included structures like Cs_0.5_Rb_0.5_Ge_0.33_Pb_0.33_Sn_0.33_(Br, Cl, I)_3_ and Cs_0.5_Rb_0.5_Ca_0.2_Ge_0.2_Pb_0.2_Sn_0.2_Sr_0.2_Br_1.5_Cl_1.5_, with entropy stabilizations of 2.54 and 2.19 kJ mol^−1^ and UCV values of 0.119 and 0.073, respectively (Fig. 6f). Their screening maps indicate that Ge, Pb, and Sn are the most promising B-site anchor cations, whereas Ca, Cd, Eu, and Sr emerge as useful secondary partners. Thus, B-site selection should distinguish between cations that preserve semiconducting functionality and those that mainly expand entropy and accessible alloy space.

The X-site offers the strongest direct control over bandgap and optical response, but it also carries the highest structural risk. Because halides occupy three positions in ABX_3_, X-site mixing can generate some of the largest entropy contributions. Muzzillo *et al.* found that alloys containing Br/Cl/I or Br/Cl/F/I on the X site reached especially large entropy-stabilization terms, but also tended to show higher UCV.^20^ For example, strong X-site participation yielded 2.77 kJ mol^−1^ for (Cs, K, Rb, Tl)Cd(Br, Cl, F, I)_3_, whereas more moderate compositions such as CsSnBrClI, CsPbBrClI, and CsGeBrClI were associated with estimated bandgaps of 1.95, 2.31, and 2.48 eV, respectively. The same study also showed that X-site compositions must be judged by entropy and mismatch together: using UCV-entropy maps, they identified a halide-perovskite phase boundary near UCV ≈ 0.04, correctly grouping 22 of 26 experimental halide-perovskite data sets, and for non-equimolar MAPb(Br, Cl, I)_3_ they reported correct classification of 52 of 56 experimental data points. This is consistent with the general experimental observation that halide migration and phase segregation become especially problematic in strongly mismatched mixed-halide systems. Accordingly, Br^−^, Cl^−^, and I^−^ define the main X-site design space for high-entropy perovskite halides, although their combinations differ markedly in structural accessibility, with Br/I mixing being generally more accessible and Cl-rich compositions, particularly Cl/I combinations, requiring much tighter structural control. The practical realization of X-site entropy engineering therefore depends strongly on the specific halide combination and on whether sufficient structural regulation is introduced to manage octahedral mismatch and halide migration.

Taken together, current computational work suggests a simple design hierarchy for future high-entropy perovskite halide engineering: A-site mixing is the most practical route for introducing stabilizing disorder with limited electronic disruption, B-site mixing is the most effective route for tuning the inorganic framework and functional properties, and X-site mixing provides the strongest leverage over optical tuning but also the greatest risk of mismatch, migration, and phase instability.

## 5. Emerging applications of high-entropy perovskite halides

High-entropy perovskite halides extend compositional engineering beyond incremental doping toward a regime where configurational disorder actively reshapes optoelectronic and electrochemical processes. Rather than simply tuning bandgaps or passivating defects, entropy-assisted compositional design can modify radiative and non-radiative pathways, carrier localization, ionic dynamics, intermediate adsorption, and redox behaviour in a site-dependent manner. However, configurational entropy is not necessarily the sole or dominant origin of these improvements, which may also arise from composition-specific changes in lattice distortion, defect chemistry, electronic structure, active-site distribution, and transport kinetics. As a result, application-level performance emerges not from a single parameter, but from the coupled control of defects, lattice stability, electronic structure, and interfacial reaction pathways. In the following, these effects are examined in light-emitting devices, photovoltaics, photodetection, imaging, and sensing systems, as well as electrochemical applications including electrocatalysis and energy storage.

### 5.1 Light-emitting devices

**Fig. 7:**
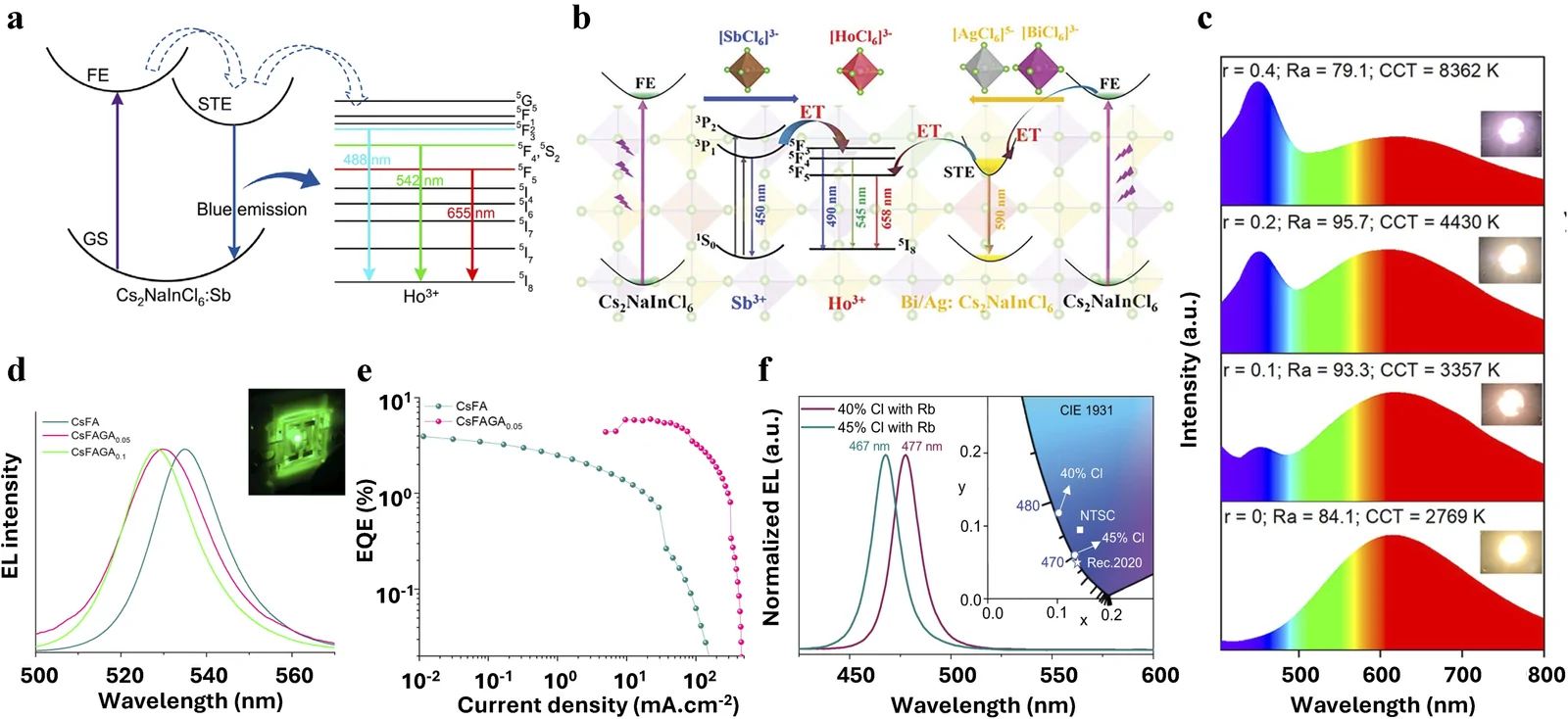
Representative examples of photophysical mechanisms and electroluminescence performances in multication and entropy-engineered perovskite emitters. (a) Energy-level diagram of Ho^3+^-doped Cs_2_NaInCl_6_:Sb^3+^ and the proposed luminescence mechanism. Reproduced with permission.^77^ Copyright 2022, Elsevier. (b) Proposed photophysical processes in Sb/Ho:Cs_2_Na_0.9_Ag_0.1_(In/Bi)Cl_6_ single-component white phosphor. Reproduced with permission.^78^ Copyright 2024, Wiley-VCH GmbH. (c) Electroluminescence spectra recorded at 100 mA and photographs of LEDs fabricated by combining a 365 nm UV-LED chip with phosphor blends of BAM : Eu and Cs_2_Ag_0.4_Na_0.6_InCl_6_ : 1% Bi, 1% Ce at different mass ratios, where r denotes the mass ratio of BAM : Eu to Cs_2_Ag_0.4_Na_0.6_InCl_6_ : 1% Bi, 1% Ce. Reproduced with permission.^79^ Copyright 2020, American Chemical Society. (d and e) Electroluminescence spectra and EQE characteristics of GA-doped triple-cation LEDs. Reproduced with permission.^83^ Copyright 2025, the Royal Society of Chemistry. (f) Electroluminescence spectra and CIE color coordinates of mixed-halide perovskite LEDs. Reproduced with permission.^84^ Copyright 2021, Springer Nature.

The development of perovskite-based light-emitting systems has its origins in optically excited architectures, particularly phosphor-converted light-emitting diodes driven by ultraviolet (UV) excitation. In these early configurations, perovskite materials were primarily employed as wavelength-conversion phosphors that absorb high-energy photons and re-emit across the visible spectrum. This approach leverages the intrinsically high photoluminescence quantum yield and defect-tolerant electronic structure of perovskite halides, especially those exhibiting self-trapped exciton (STE) emission. Low-dimensional and double perovskite systems have demonstrated broadband emission spanning the visible region, enabling single-component white phosphors. For example, STE-based emission in layered perovskites covers approximately 400–800 nm, providing continuous spectral output suitable for white light generation. In addition, rare-earth-doped double perovskites such as Ho^3+^ incorporated Cs_2_(Na, Ag)InCl_6_ exploit energy transfer from host STE states to dopant ions, producing combined broadband and line emission under 365 nm excitation and improving colour rendering performance (Fig. 7a).^77^ Similarly, lead-free double perovskites such as Cs_2_Ag_0.4_Na_0.6_InCl_6_:Bi, Ce exhibit near-unity internal quantum efficiency (∼98.6%) and have been successfully integrated with UV LED chips to achieve high colour rendering index (>95) in white light emission (Fig. 7b).^78^ More recently, high-entropy perovskite halides such as (TEA)_2_(Zr, Te, Hf, Sn, Pt)Cl_6_ have extended this concept by introducing multicomponent disorder, enabling broadband absorption (250–450 nm) and efficient emission through energy transfer among multiple octahedral units under UV excitation.^56^ Collectively, these results demonstrate that perovskites can achieve highly efficient light emission under optical excitation through exciton localization and energy transfer, but do not yet address the requirements for electrically driven operation.

Despite these advances, UV-driven phosphor systems are fundamentally limited by their reliance on external optical excitation. Wang *et al.* showed LEDs incorporating a 365 nm UV chip and blends of BAM:Eu with Cs_2_Ag_0.4_Na_0.6_InCl_6_:Bi, Ce phosphor at different mass ratios show characteristic PL spectra (Fig. 7c).^79^ In these systems, light emission is governed by photoluminescence processes rather than direct carrier recombination, leading to inherent inefficiencies associated with photon conversion, reabsorption losses, and device complexity arising from the need for separate excitation sources. More importantly, the absence of electrical carrier injection prevents full utilization of the unique electronic properties of perovskites, including their high radiative recombination rates, defect tolerance, and tuneable band-edge states. These limitations motivate the transition toward PeLEDs, where emission arises from the recombination of injected electrons and holes within the emissive layer.^80^ This challenge directly motivates the need for strategies that can simultaneously suppress non-radiative recombination and stabilize carrier dynamics under electrical bias. Representative light-emitting device architectures and their corresponding optical performances for high-entropy perovskite halides are summarized in Table 1.

**Table 1:** Representative light-emitting sevice architectures and optical performances of high, medium, low-entropy perovskite halides

	Perovskites	Synthetic methods	EL/PL peak (nm)	Δ*S*_config_				PLQY (%)	Device structure	Ref.
				A-site	B-site	X-site	Total (entropy class)			
Phosphor based LEDs	Cs(Mn_*x*_Ni_1−*x*/4_Ca_1−*x*/4_Mg_1−*x*/4_Pb_1−*x*/4_)Cl_3_	Ball milling	585	0	1.61	0	1.61 (high)	—	365 nm InGaN chip + PeNCs/resin coating	65
	Cs(Pb_1/6_Mn_1/6_Ni_1/6_Zn_1/6_Yb_1/6_Ce_1/6_)Cl_3_	Ball milling	600	0	1.79	0	1.79 (high)	—	365 nm LED chip + UV-curable epoxy resin/PeNCs coating	85
	Cs(Na_0.2_Bi_0.2_Mn_0.2_Ni_0.2_Zn_0.2_)Cl_3_	Ball milling	585	0	1.61	0	1.61 (high)	5.02	—	70
	Cs(PbSnSrMnZn)Br_3_	LARP	525	0	1.61	0	1.61 (high)	≈98	Single-conversion-layer LED with 360 nm chip	18
	(TEA)_2_(Zr_0.18_Te_0.22_Hf_0.2_Sn_0.3_Pt_0.1_)Cl_6_	Colloidal synthesis	580	0	1.56	0	1.61 (high)	—	—	56
	Cs_2_(NaAg)(InSbHoErBi)_1_Cl_6_	Colloidal synthesis	550	0	B-0.69	0	2.30 (high)	8.7	—	31
					B′-1.61					
	Cs(Pb_0.2_Mn_0.2_Ni_0.2_Zn_0.2_Yb_0.2_)Cl_3_	Ball milling	600	0	1.61	0	1.61 (high)	46	—	63
	Cs(Pb_0.2_Mn_0.2_Ni_0.2_Zn_0.2_Cd_0.2_)Br_3_/Cs(Pb_0.2_Mn_0.2_Ni_0.2_Zn_0.2_Cd_0.2_)2Br_5_	Ball milling	600	0/0	1.61/1.61	0/0	1.61 (high)	24.5	—	86
	Cs(Pb_0.72_Mn_0.14_Ni_0.14_)Br_3_	Ball milling	515	0	0.79	0	0.79 (low)	14.26	—	87
	MA(PbMgZnCd)Br_3_	Postsynthetic cation-exchange routes	504	0	1.39	0	1.39 (medium)	95	365 nm UV-LED chip + phosphor coating (with BAM:Eu for WLED)	72
	Cs_2_Ag_0.40_Na_0.60_InCl_6_:Bi, Ni	Colloidal synthesis	570	0	0.67	0	0.69 (low)	—	365 nm UV LED chip + phosphor coating	79
	Cs_2_(Na,Ag)InCl_6_:Bi, Ho^3+^	Hydro-thermal	400–800 (white)	0	0.69	0	0.69 (low)	60.49	ITO/NiO_*x*_NC/PVK:PVP/PeNCs/TPBi/LiF/Al	71
PeLEDs	Cs_0.7_(FA_0.2_Rb_0.1_)Pb(Br_0.6_Cl_0.4_)_3_	Vapor-assisted crystallization	477	0.80	0	0.67	2.82 (high)	37	ITO/ZnO/PEI/PeNCs/TCTA/MoO_*x*_/Ag	81
	GA_0.28_Cs_0.72_Pb_0.40_Ge_0.25_Cd_0.05_I_3_	Colloidal synthesis	632	0.59	0.88	0	1.82 (high)	≈100	ITO/PEDOT:PSS/poly-TPD/PeNCs/TPBi/LiF/Al	73
	CsCd_0.1_Pb_0.8_Sr_0.1_Br_3_	Ultrasonic-assisted strategy	∼510–530	0	0.64	0	0.64 (low)	—	ITO/PEDOT:PSS:Nafion/TFB/PeNCs/TPBi/Liq./Al	16
	(Cs_0.1_FA_0.9_)_0.95_GA_0.05_PbBr_3_	Postsynthetic cation-exchange routes	525	0.51	0	0	0.51 (low)	91.8		83

In PeLEDs, electroluminescence is governed by charge injection, transport, and radiative recombination dynamics. Efficient device operation requires that injected carriers recombine radiatively before being lost through non-radiative pathways or leakage, placing stringent constraints on defect density, compositional uniformity, and charge balance. Unlike photovoltaic systems, where carriers can be extracted before recombination, PeLEDs rely entirely on radiative recombination, making them particularly sensitive to trap states and local heterogeneity.^81^ As a result, suppressing non-radiative recombination and stabilizing carrier dynamics under electrical bias are central challenges in PeLED design.

Within this context, entropy engineering provides a coherent framework for regulating these challenges through site-dependent disorder. Building on the structure–property relationships discussed above, A-site entropy primarily governs lattice stability and ion migration, whereas B-site entropy directly modifies the band-edge electronic structure and radiative recombination pathways. From a bulk electronic perspective, B-site entropy engineering plays a dominant role in enhancing electroluminescence efficiency. In multicomponent B-site-alloyed NCs such as CsCd_0.1_Pb_0.8_Sr_0.1_Br_3_, which emit in the green spectral region (∼510–530 nm), multicomponent B-site incorporation increases compositional disorder and raises defect formation energies, suppressing deep trap states that act as non-radiative recombination centres. In addition, band-edge reconstruction induced by compositional disorder can generate shallow or localized electronic states that favour radiative recombination, further enhancing emission efficiency. As a result, an EQE exceeding 20%, with a reported value of 22.2%, has been achieved, compared to ∼10–15% for conventional CsPbBr_3_ systems.^16^

In contrast, A-site entropy engineering contributes primarily to electroluminescence stability by regulating lattice dynamics and ionic transport. In multication systems such as Cs_*x*_(MA_0.17_FA_0.83_)_1−*x*_PbBr_3_, increased compositional disorder has been associated with ion migration and stabilizes the lattice under electrical injection.^82^ This effect is particularly important in PeLED operation, where ion migration can dynamically generate non-radiative recombination centres and shift the recombination zone, leading to spectral instability and efficiency loss. By suppressing these processes, multication A-site engineering helps maintain a stable recombination zone and sustain EL efficiency during operation. Related triple-cation Cs–FA–GA quantum dots further demonstrate that compositional complexity can suppress surface defects and improve radiative recombination, leading to PLQY values up to 91.8% and PeLED EQEs approaching 5.9% (Fig. 7d and e).^83^

The combined roles of A-site and B-site entropy become especially critical in wide-bandgap blue emitters. In mixed-halide systems such as CsFAPb(Br_1−*x*_Cl_*x*_)_3_, emission can be tuned across the blue spectral region (∼451–490 nm), but compositional heterogeneity leads to bandgap fluctuations and defect-rich domains that act as carrier trapping centres. Studies on Cs–FA–Rb Br/Cl perovskites show that even minor heterogeneity significantly increases non-radiative recombination under electrical injection.^81^ Multication alloying suppresses such clustering, leading to a more homogeneous emissive phase and improved radiative recombination efficiency. Correspondingly, blue PeLEDs with EQEs up to ∼11% at ∼477 nm and ∼5.5% at ∼467 nm have been demonstrated with improved spectral stability (Fig. 7f).^84^ Overall, entropy engineering enables the translation of high photoluminescence efficiency in optically excited phosphor systems into efficient electroluminescence in electrically driven devices by aligning material properties with device-level recombination requirements. By increasing defect formation energy, suppressing compositional heterogeneity, and stabilizing recombination pathways, entropy-assisted compositional design provides a unified framework for controlling emission efficiency, spectral stability, and device operation in PeLEDs.^64^

For light-emitting applications, B-site engineering is most directly associated with defect energetics and radiative recombination, whereas A-site mixing primarily supports lattice and spectral stability. Combined A/B-site regulation is therefore most advantageous when high emission efficiency and operational stability must be achieved simultaneously, while mixed-halide X-site tuning remains constrained by compositional heterogeneity and halide migration.

### 5.2 Photovoltaics

#### 5.2.1 Entropy engineering in single-junction photovoltaics

**Fig. 8:**
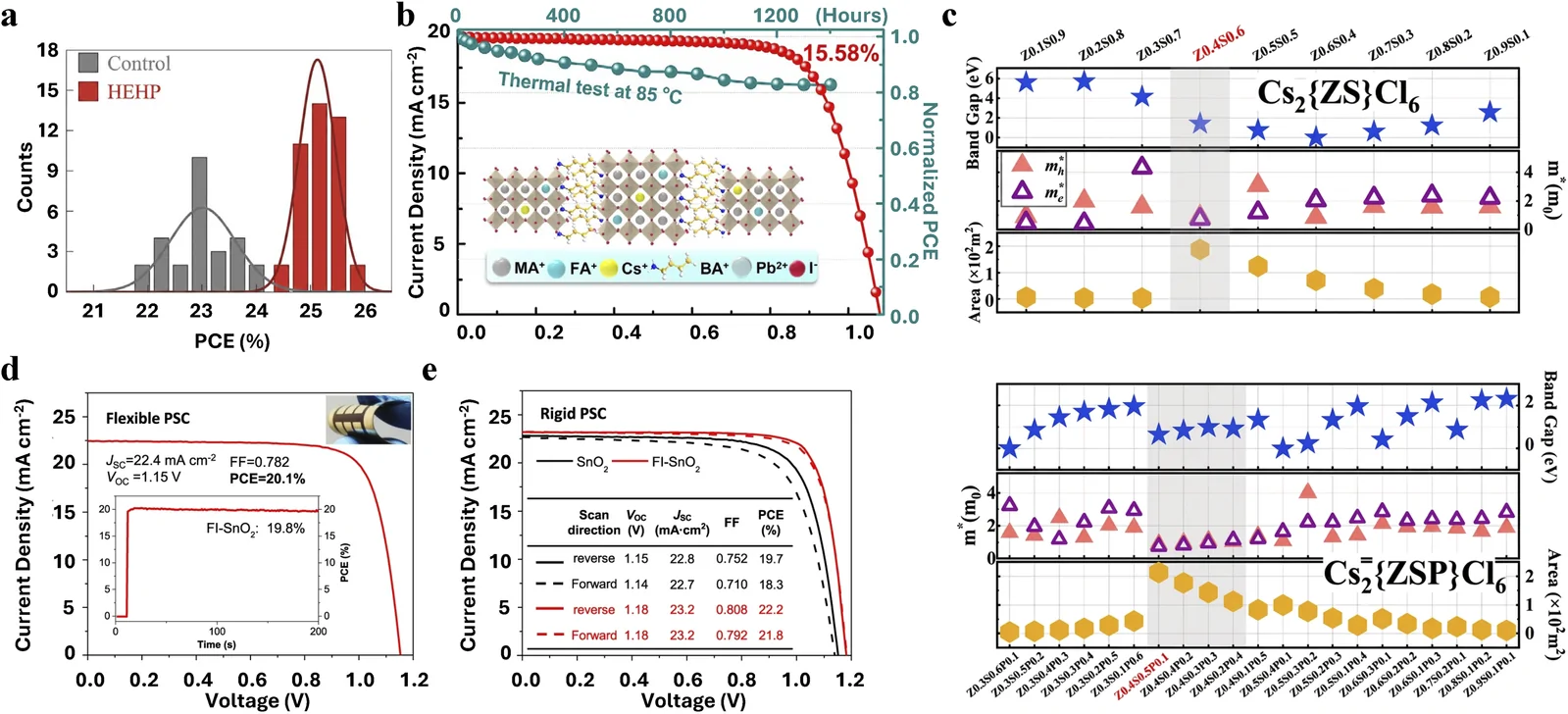
Representative examples of compositional and interfacial engineering strategies for high-performance perovskite solar cells. (a) PCE distribution of inverted perovskite solar cells fabricated with and without and high-entropy hybrid perovskite. Reproduced with permission.^4^ Copyright 2024, Springer Nature. (b) *J*–*V* characteristics and normalized PCE retention under thermal stressing at 85 °C, highlighting the enhanced thermal stability and efficiency of the triple-A-site-cation RP perovskite solar cell; (inset) schematic illustration of the intralayer A-site compositional engineering strategy. Reproduced with permission.^91^ Copyright 2019, American Chemical Society. (c) Calculated bandgaps (upper panel), carrier effective masses (middle panel), and absorption spectra area (lower panel) of Cs_2_(ZS)Cl_6_ and Cs_2_(ZSP)Cl_6_ materials with the R^2^SCAN functional, where Z = Zr, S = Sn, P = Pt. Reproduced with permission.^92^ Copyright 2025, Elsevier. (d) *J*–*V* curve and MPP PCE output of the champion flexible PSC based on FI-SnO_2_ ETL; (inset) photograph of the flexible PSC device. (e) *J*–*V* curves of rigid PSCs based on FI-SnO_2_ and pristine SnO_2_ ETLs under forward and reverse scans; (inset table) extracted photovoltaic parameters. Reproduced with permission.^90^ Copyright 2021, Springer Nature.

The performance of PSCs is governed by the interplay between defect energetics, phase stability, and electronic structure, all of which are strongly influenced by compositional complexity. In contrast to single-cation systems, multication and compositionally complex perovskites exhibit emergent behaviour that enables simultaneous optimization of efficiency and stability.^8,88^ The suppression of non-radiative recombination has been identified as a key factor in achieving high device performance. In multication hybrid perovskites such as (Fa_0.98_MA_0.02_)_0.95_Cs_0.05_Pb(I_0.95_Br_0.05_)_3_, PCEs as high as 25.7% have been reported, accompanied by a high fill factor of 85.2% (Fig. 8a).^4^ Such performance is generally attributed to reduced trap-assisted recombination, which arises from improved lattice stability and compositional homogeneity. The introduction of multiple A-site cations increases configurational entropy, which may contribute to higher defect formation energies and the suppression of deep trap states together with composition-specific structural and chemical effects. As a result, carrier lifetime is extended and non-radiative losses are reduced.^62,89^

Beyond reducing non-radiative recombination, mixed A-site compositions can improve the homogeneity and operational stability of the photoactive phase. For example, FA_0.83_Cs_0.17_PbI_2.7_Br_0.3_ exhibited a balanced photovoltaic response with *V*_oc_ ≈ 1.14 V, *J*_sc_ ≈ 23.2 mA cm^−2^, FF ≈ 80%, leading to a PCE of 21.1%.^10^ This behaviour is commonly associated with the suppression of phase segregation through mixed A-site cations. Increased configurational entropy may contribute to lowering the free energy of the mixed phase, together with changes in lattice dimensions, defect chemistry, and compositional homogeneity, thereby improving structural stability under operating conditions. Similarly, Cs_0.04_(FA_0.84_MA_0.16_)_0.96_Pb(I_0.84_Br_0.16_)_3_ achieves a relatively high open-circuit voltage of 1.18 V, together with a PCE of 22.2%, which has been linked to reduced energetic disorder and suppressed non-radiative recombination arising from improved compositional uniformity and lattice stabilization.^90^ Structural stabilization must nevertheless be balanced with efficient carrier transport. The layered perovskite BA_2_(MA_0.76_FA_0.19_Cs_0.05_)_3_Pb_4_I_13_ showed improved structural stability but lower PCE of 15.58%, which was associated with transport limitations and recombination barriers arising from reduced dimensionality. Consistent with this interpretation, Jiang *et al.* presents the *J*–*V* characteristics and normalized PCE retention under thermal stressing at 85 °C for a triple-A-site-cation Ruddlesden–Popper (RP) perovskite solar cell (Fig. 8b).^91^ These observations indicate that achieving optimal performance requires a balance between structural stability and efficient charge transport.

B-site compositional tuning provides a complementary route for regulating the electronic structure of photovoltaic absorbers. Hu *et al.* used computational screening of binary and ternary Cs_2_MCl_6_ derivatives to identify composition-dependent changes in bandgap, carrier effective mass, and optical absorption (Fig. 8c). Guided by these trends, they designed the six-component vacancy-ordered Cs_2_(Zr_0.18_Sn_0.36_Te_0.27_Hf_0.09_Re_0.05_Pt_0.05_)Cl_6_, which was calculated to have a bandgap of 1.7 eV. Semiconductor-device simulations further predicted a PCE of 30.35% in an FTO/SnO_2_/perovskite/CuSCN/Au configuration. The related five-component Cs_2_(Zr_0.18_Sn_0.36_Te_0.27_Hf_0.09_Pt_0.10_)Cl_6_ exhibited a lower simulated PCE of 17.67%, indicating that photovoltaic performance depends strongly on the identities and ratios of the constituent elements rather than on compositional complexity alone.^92^

Material-level stabilization alone does not guarantee efficient photovoltaic operation because charge extraction and interfacial recombination remain strongly governed by transport-layer quality and energy-level alignment. PSCs employing FI-SnO_2_ as the electron-transport layer exhibit improved *J*–*V* behaviour, enhanced maximum-power-point output, and more favourable photovoltaic parameters than devices based on pristine SnO_2_ (Fig. 8d and e).^90^ Compositional stabilization of the absorber and targeted interface engineering should therefore be regarded as complementary strategies for achieving efficient and durable single-junction PSCs. Representative photovoltaic device structures and performances of high-entropy perovskite halides are summarized in Table 2.

**Table 2:** Representative photovoltaic device structures and performances of high, medium, low-entropy perovskite halides

Perovskites	Bandgap (eV)	*V*_oc_ (V)	*J*_sc_ (mA cm^−2^)	FF (%)	PCE (%)	Δ*S*_config_				Device structure	Ref.
						A-site	B-site	X-site	Total (entropy class)		
Cs_2_(Zr_0.18_Sn_0.36_Te_0.27_Hf_0.09_Re_0.05_Pt_0.05_)Cl_6_ (predicted)	1.7	—	—	—	30.35	0	1.55	0	1.55 (high)	FTO/SnO_2_/PeNCs/CuSCN/Au	92
Cs_2_(Zr_0.18_Sn_0.36_Te_0.27_Hf_0.09_Pt_0.1_)Cl_6_ (predicted)	1.21	—	—	—	17.67	0	1.48	0	1.48 (medium-high)	FTO/SnO_2_/PeNCs/CuSCN/Au	92
(FA_0.98_MA_0.02_)_0.95_Cs_0.05_Pb(I_0.95_Br_0.05_)_3_	1.51	1.17	25.8	85.2	25.7	0.29	0	0.20	0.89 (low)	ITO/SAM/PeNCs/LiF/C60/BCP/Ag	4
Cs_0.04_(FA_0.84_MA_0.16_)_0.96_Pb(I_0.84_Br_0.16_)_3_	1.7	1.18	23.2	80.8	22.2	0.59	0	0.44	1.91 (high)	FTO/FI-SnO_2_/PeNCs/Spiro-OMeTAD/Au	90
GA_0.015_Cs_0.046_MA_0.152_FA_0.787_Pb(I_0.815_Br_0.185_)_3_	1.62	1.18	23.6	—	20.96	0.68	0	0.48	2.12 (high)	FTO/compact-TiO_2_/mesoporous TiO_2_/PeNCs/spiro-OMeTAD/Au	89
BA_2_(MA_0.76_FA_0.19_Cs_0.05_)_3_Pb_4_I_13_	1.59	1.08	19.67	73.3	15.58	0.67	0	0	2.02 (high)	ITO/MoO_3_/PEDOT:PSS/RP PeNCs/PC_61_BM/BCP/Ag	91
Rb_0.05_Cs_0.05_FA_0.75_MA_0.15_Pb(I_0.95_Br_0.05_)_3_	—	1.103	23.8	78	20.6	0.80	0	0.20	1.40 (medium)	ITO/compact TiO_2_/mesoporous TiO_2_/PeNCs/PTAA/Au	93
Eu^3+^-modified (FA,MA,Cs)Pb(I, Br)_3_(Cl)	1.55	1.153	—	77.8	21.52	—	—	—	—	ITO/SnO_2_/PeNCs/spiro-OMeTAD/Au	94
FA_0.83_Cs_0.17_PbI_2.7_Br_0.3_	1.63	1.14	23.2	80	21.1	0.46	0	0.33	1.43 (medium)	FTO/SnO_2_/PeNCs/spiro-OMeTAD/Au	95
Cs_0.25_FA_0.75_Pb(Br_0.20_I_0.80_)_3_	1.68	1.1	19.4	81	17.4	0.56	0	0.50	2.06 (high)	ITO/PTAA/PeNCs/C60/BCP/Ag	10
Cs_0.05_(FA_0.83_MA_0.17_)_0.95_Pb(I_0.83_Br_0.17_)_3_	—	1.14	23.6	77	20.8	0.63	0	0.46	2.00 (high)	FTO/compact TiO_2_/mesoporous TiO_2_/PeNCs/spiro-OMeTAD/Au	96
FA_0.80_MA_0.15_Rb_0.05_PbI_2.55_Br_0.45_	—	1.168	23.2	74.7	20	0.61	0	0.42	1.88 (high)	FTO/compact-TiO_2_/mesoporous-TiO_2_/PeNCs/spiro-MeOTAD/Au	97
Rb_0.05_FA_0.75_MA_0.15_Cs_0.10_PbI_2_Br	1.73	1.13	19.4	70	15.4	0.89	0	0.64	2.80 (high)	FTO/compact-TiO_2_/mesoporous TiO_2_/PeNCs/PTAA/Au	46
Cs_0.05_(MA_0.17_FA_0.83_)_0.95_Pb(I_0.83_Br_0.17_)_3_	—	1.132	22.96	74.8	∼20	0.63	0	0.46	2.00 (high)	Glass/FTO/compact TiO_2_/Li-doped mesoporous TiO_2_/PeNCs/spiro-OMeTAD/Au	98

#### 5.2.2 Wide-bandgap absorbers and tandem photovoltaics

Wide-bandgap perovskite absorbers with bandgaps in the approximate range of 1.7–1.8 eV are central to perovskite–silicon and all-perovskite tandem photovoltaics, where they serve as top cells paired with lower-bandgap bottom absorbers. Bandgaps near 1.67 eV are commonly targeted for perovskite–silicon tandems and are generally obtained by increasing the Br fraction in mixed I/Br perovskites.^99^ However, compositions containing more than approximately 20% Br are particularly susceptible to photoinduced halide segregation, which compromises their spectral and photovoltaic stability under illumination.^100^

The entropy–enthalpy framework introduced in Section 2.3 provides a thermodynamic basis for the reversible nature of this process. In the dark, the compositional free energy of a mixed-halide phase reflects competition between mixing enthalpy and the stabilizing contribution of configurational mixing entropy. Under illumination, the electronic free energy of photocarriers must also be considered. Stochastic compositional fluctuations generate I-rich regions with bandgaps below that of the parent phase, allowing photocarriers to lower their free energy by accumulating within these regions. Carrier accumulation promotes inward diffusion of I and outward diffusion of Br, resulting in nucleation and growth of segregated domains. When illumination is removed, the photocarrier contribution disappears, and configurational entropy and concentration gradients favour restoration of the mixed halide distribution.^66,100^ The onset and kinetics of segregation are additionally influenced by temperature, carrier density, defects, and ion migration, while lattice strain has also been proposed to affect the nucleation and stability of segregated domains. For MAPbBr_1.5_I_1.5_, Elmelund *et al.* obtained activation energies of 28.9 kJ mol^−1^ for photoinduced segregation and 53.5 kJ mol^−1^ for dark remixing. The threshold illumination intensity increased from approximately 30 µW cm^−2^ at 23 °C to 100 µW cm^−2^ at 90 °C because thermally activated remixing became increasingly competitive at higher temperatures.^101^ Exchange between I^−^ and Br^−^ is further facilitated by halide vacancies, indicating that the thermodynamic driving force and kinetic migration pathways must be considered together.

Prolonged illumination experiments have clarified how segregation affects actual device operation and how A-site alloying can moderate this behaviour. Datta *et al.* investigated the 1.75 eV absorber FA_0.66_MA_0.34_Pb(I_0.67_Br_0.33_)_3_ under illumination for approximately 28 h.^100^ The formation of isolated I-rich domains produced red-shifted and intensified photoluminescence as carriers accumulated and recombined within the lower-bandgap regions. Rather than causing a dominant loss in *V*_oc_, these domains obstructed carrier collection and produced a pronounced decrease in *J*_sc_. Photoluminescence, photovoltaic performance, and sub-bandgap defect characteristics were largely restored after prolonged storage in the dark, where removal of the photocarrier free-energy contribution allowed configurational entropy and concentration gradients to favour restoration of the mixed-halide distribution. Partial substitution of FA^+^ and MA^+^ with Cs^+^ delayed illumination-induced degradation and reduced both the initial density and light-induced growth of sub-bandgap defects, although segregation was not completely prevented. A unified thermodynamic model provided an additional explanation for the stabilizing role of Cs incorporation. Partial Cs substitution reduced the bandgap difference between the homogeneous parent phase and the nucleated I-rich phase. Because this difference determines the electronic free-energy gain associated with carrier funneling, decreasing it raises the photocarrier-density threshold required for segregation.^66^ Together, these studies suggest that A-site alloying may influence photostability through coupled changes in configurational entropy, lattice dimensions, octahedral distortion, defect formation, and the electronic contrast between the mixed and segregated phases rather than through entropy alone.

Although not a formally high-entropy composition, triple-halide alloying provides an important precedent for using broader compositional control to stabilize a tandem-compatible absorber. Xu *et al.* reduced the Br fraction of Cs_0.25_FA_0.75_Pb(I_1−*x*_Br_*x*_)_3_ from 20% to 15% and introduced 2–5 mol% MAPbCl_3_ into Cs_0.25_FA_0.75_Pb(I_0.85_Br_0.15_)_3_.^99^ Bulk Cl incorporation increased the bandgap from approximately 1.63 eV to above 1.67 eV while retaining a single-phase lattice with a reduced lattice parameter and a uniform halide distribution. The 1.67 eV triple-halide film showed nearly twofold increases in photocarrier lifetime and mobility relative to the I/Br control, together with a lower defect density. No low-energy photoluminescence peak developed under conditions that produced clear segregation in the control, and light-induced segregation remained strongly suppressed even at excitation intensities equivalent to 100 suns. The strategy also reduced the *V*_oc_ deficit by approximately 100 mV and increased the wide-bandgap cell PCE from approximately 18% to 20.3%. Semi-transparent top cells retained more than 96% of their initial efficiency after 1000 h of maximum-power-point operation at 60 °C and more than 97% after 500 h at 85 °C. Integration with a silicon bottom cell produced a 27% efficient two-terminal monolithic tandem with an area of 1 cm^2^. These results demonstrate how coordinated A-site and X-site compositional control can regulate the bandgap, lattice dimensions, defect density, carrier transport, and halide stability.

A complementary conventional strategy is to regulate composition locally at the absorber interface rather than throughout the bulk. Chai *et al.* employed the 1.68 eV absorber FA_0.65_MA_0.20_Cs_0.15_Pb(I_0.8_Br_0.2_)_3_ together with Pb(SCN)_2_ addition and MACl post-treatment. Pb(SCN)_2_ improved crystallization but generated excess PbI_2_ at grain boundaries, which reacted with MACl to form a near-surface MAPbI_3−*x*_Cl_*x*_ graded heterojunction. The resulting compositional gradient and compressive strain improved energy-level alignment while suppressing ion migration, non-radiative recombination, and halide.^102^ The optimized wide-bandgap PSC achieved a PCE of 20.30% and a steady-state PCE of 19.24% during 300 s of maximum-power-point operation. The unencapsulated device retained 93.5% of its initial efficiency after 1200 h of ambient storage and 91.0% after heating at 85 °C for 8 h. Combining the wide-bandgap cell with a TOPCon silicon device yielded 30.91% efficient four-terminal tandem.

The tandem studies discussed above rely on mixed-cation, triple-halide, additive, and interfacial strategies rather than formally high-entropy compositions. Nevertheless, they demonstrate that compositional complexity can regulate key thermodynamic and kinetic factors associated with wide-bandgap instability, including mixed-phase free energy, defect formation, halide migration, and the energetic driving force for segregation.^66,100^ Applying high-entropy design to wide-bandgap absorbers therefore represents a natural and important direction for tandem photovoltaics, although it should be regarded as an emerging strategy rather than an established solution.

### 5.3 Photodetectors, imaging, and sensing

High-entropy perovskite halides are emerging as a versatile platform for photodetectors, imaging, and sensing, because entropy-driven disorder enables efficient signal generation, modulation, and attenuation across multiple detection modalities. Rather than relying solely on bandgap tuning, these systems leverage lattice distortion and carrier localization to directly enhance how external stimuli are converted into measurable outputs.

**Fig. 9:**
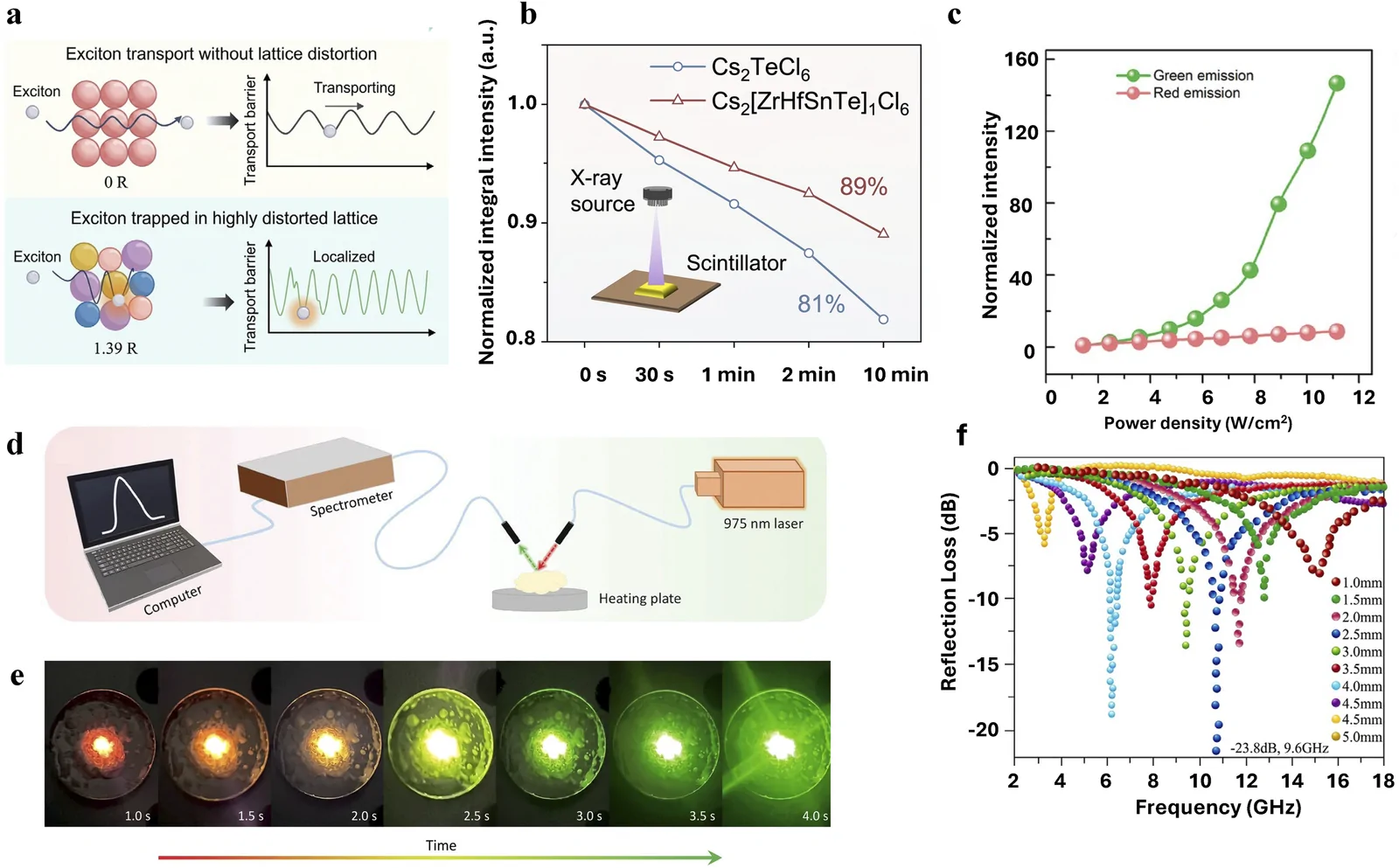
Representative functional applications enabled by entropy engineering in metal perovskite halides. (a) Schematic illustration of exciton transport and recombination in Cs_2_MCl_6_ with configurational entropies of 0 and 1.39*R*, showing that high-entropy induces pronounced lattice distortion, enhanced exciton transport barriers, and exciton localization. (b) Normalized X-ray excited luminescence (XEL) integral intensity of Cs_2_TeCl_6_ and Cs_2_[ZrHfSnTe]Cl_6_ after X-ray irradiation from 30 s to 10 min reproduced with permission.^103^ Copyright 2025, American Chemical Society. (c) Power-dependent normalized emission intensities and double-logarithmic plots of pump power *versus* emission intensity for Cs_3_GdO_*x*_Cl_6−*x*_:0.2Yb/0.02Er crystals. (d) Schematic illustration of the temperature-dependent spectral testing system. (e) Time-dependent photographs showing the colour evolution of Cs_3_GdO_*x*_Cl_6−*x*_:0.2Yb/0.02Er under 975 nm excitation. Reproduced with permission.^104^ Copyright 2024, Wiley-VCH GmbH. (f) Reflection loss (RL) spectra of Cs(Pb, Fe, Co, Ni, Mn)Br_3_ at different thicknesses, showing strong electromagnetic-wave attenuation, with the maximum absorption centred at 10.2 GHz. Reproduced with permission.^105^ Copyright 2024, Elsevier.

In radiation detection and imaging, the key requirement is efficient conversion of high-energy input into optical signals with minimal loss. Tang *et al.* demonstrated that entropy-engineered Cs_2_MCl_6_ scintillators significantly improve this conversion efficiency by inducing lattice distortion within [MCl_6_]^2−^ octahedra, which increases exciton localization and suppresses non-radiative recombination.^103^ Increasing configurational entropy induces pronounced lattice distortion, raises exciton-transport barriers, and promotes exciton localization (Fig. 9a). As a result, the photoluminescence quantum yield increases by ∼8-fold and emission intensity by ∼17-fold compared to the single-component system. Consistent with this mechanism, the normalized X-ray-excited luminescence of Cs_2_TeCl_6_ and Cs_2_[ZrHfSnTe]_1_Cl_6_ under continuous X-ray irradiation further supports the entropy-enhanced radiative process (Fig. 9b). This enhancement directly translates into device-level imaging performance, where a low X-ray detection limit of 50.3 nGy s^−1^ is achieved, meaning that extremely weak radiation signals can be reliably detected. At the same time, the spatial resolution exceeds 20 lp mm^−1^, indicating that localized exciton recombination enables high-fidelity image reconstruction. These results establish that entropy-induced exciton localization can be advantageous for scintillation-based imaging by suppressing exciton migration and promoting radiative recombination, thereby improving imaging sensitivity and spatial resolution. In optical sensing, high-entropy perovskites enable signal transduction through spectral modulation rather than simple intensity change. Hu *et al.* reported that entropy-induced disorder modifies the local crystal field and enables additional splitting of rare-earth 4f energy levels in upconversion perovskites. This leads to a temperature-dependent redistribution of emission pathways, where the green emission (∼525 nm) increases by ∼62 times between 25 and 150 °C, while the red emission (∼660 nm) remains nearly constant.^104^ This sensing behaviour is accompanied by characteristic excitation-power-dependent emission, a defined temperature-dependent spectral testing configuration, and rapid time-dependent colour evolution under 975 nm excitation (Fig. 9c and e). The significance of this behaviour is that sensing information is encoded in wavelength-dependent emission changes, enabling more robust, multi-channel detection than single-intensity signals. Furthermore, rapid colour switching within a few seconds under 975 nm excitation indicates fast carrier relaxation dynamics, which is essential for real-time sensing applications.

In electromagnetic sensing and interference detection, entropy engineering enhances signal attenuation through coupled dielectric and magnetic responses. Chang *et al.* showed that introducing multiple transition metals into CsPbBr_3_ generates synergistic disorder that increases both magnetic polarization and dielectric loss.^105^ This results in a minimum reflection loss of −75 dB at 10.2 GHz, corresponding to near-complete absorption of incident electromagnetic waves, and an effective bandwidth of 8.8 GHz, indicating stable operation over a wide frequency range (Fig. 9f). These metrics demonstrate that entropy-driven structural disorder can convert incident electromagnetic signals into dissipated energy with high efficiency, making these materials suitable for sensing electromagnetic interference and signal shielding environments.

Taken together, high-entropy perovskite halides enable three distinct but complementary sensing functions. Radiation detection benefits from enhanced radiative recombination and low detection limits. Optical sensing exploits entropy-induced spectral tunability for multi-channel signal encoding. Electromagnetic sensing leverages disorder-enhanced attenuation for broadband signal absorption. Across all cases, the unifying mechanism is the conversion of configurational disorder into controlled carrier localization and energy dissipation pathways, establishing entropy engineering as a general design principle for advanced photodetectors, imaging systems, and sensing technologies.

### 5.4 Electrochemical applications

**Fig. 10:**
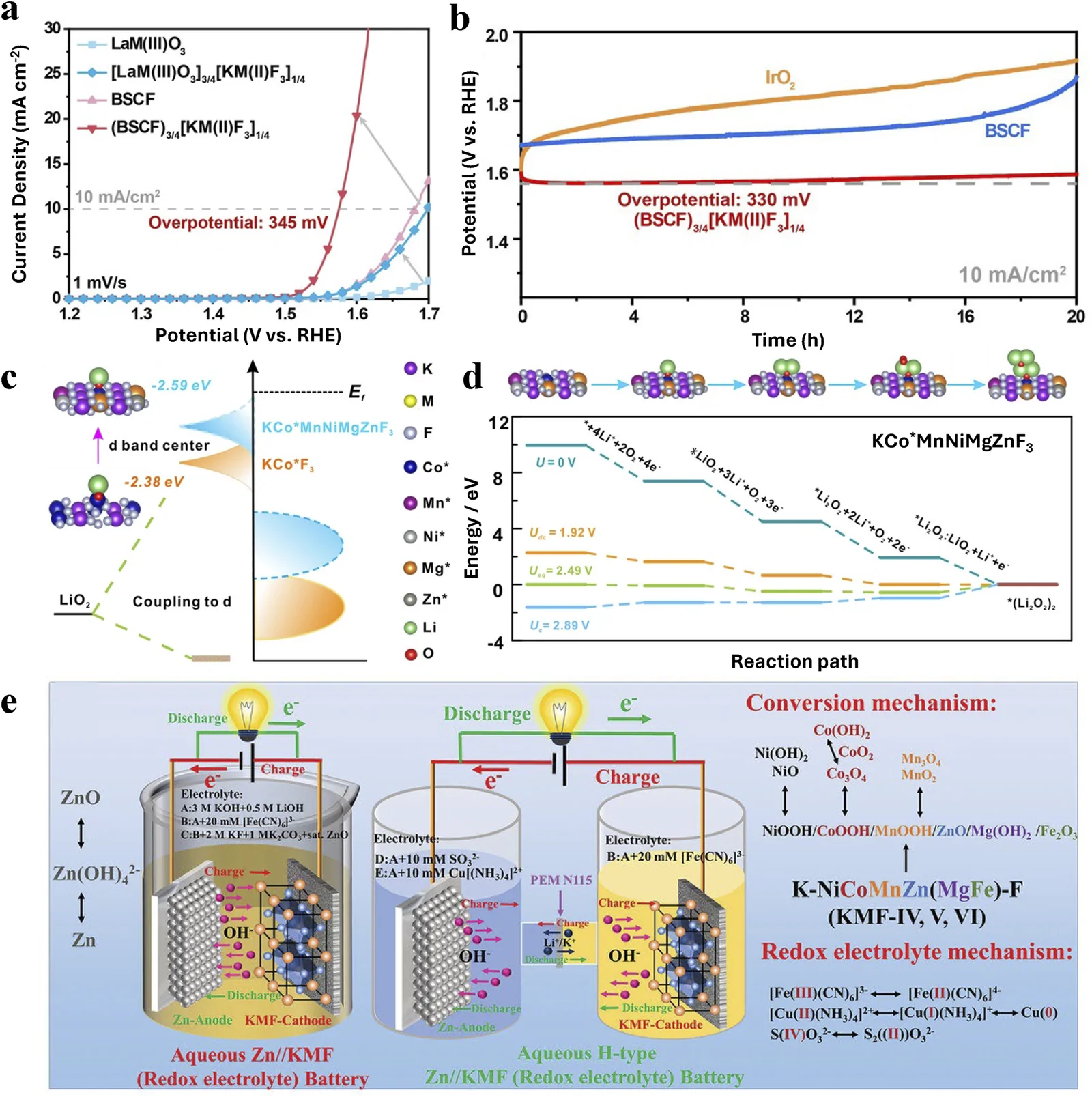
Representative electrochemical applications and reaction mechanisms of high-entropy and multicomponent perovskite fluorides. (a) OER polarization curves of LaM(iii)O_3_, BSCF, [LaM(iii)O_3_]_3/4_[KM(ii)F_3_]_1/4_, and (BSCF)_3/4_[KM(ii)F_3_]_1/4_ in 1 M KOH (b) chronopotentiometric stability of commercial IrO_2_, BSCF, and (BSCF)_3/4_[KM(ii)F_3_]_1/4_ at 10 mA cm^−2^. Reproduced with permission.^107^ Copyright 2021, Wiley-VCH GmbH. (c) Schematic illustration of d–p orbital hybridization between Co* active sites and the LiO_2_ intermediate during the ORR in KCoF_3_ and KCoMnNiMgZnF_3_-HEC. (d) Calculated Gibbs free-energy for the ORR and pathway on the Co* site of KCoMnNiMgZnF_3_-HEC. Reproduced with permission.^109^ Copyright 2023, Springer Nature. (e) Schematic illustration of the operating principles of Zn//KMF-IV, Zn//KMF-V, and Zn//KMF-VI aqueous batteries during charge and discharge. Reproduced with permission.^115^ Copyright 2023, Wiley-VCH GmbH.

Beyond the Cl-, Br-, and I-based optoelectronic systems discussed above, high-entropy perovskite fluorides have been investigated for electrochemical energy conversion and storage. Across these applications, high-entropy design can stabilize multication perovskite phases, create chemically diverse local environments, regulate transition-metal electronic states, and alter ion-transport and redox pathways. Wang *et al.* first demonstrated entropy-assisted formation of single-phase perovskite fluorides containing five different B-site cations, including compositions for which the corresponding binary combinations were difficult to stabilize as homogeneous phases.^106^ When applied to alkaline oxygen evolution reaction (OER), K(Mg_0.2_Mn_0.2_Fe_0.2_Co_0.2_Ni_0.2_)F_3_ exhibited lower polarization resistance and faster reaction kinetics than the individual KMF_3_ fluorides, despite containing relatively small fractions of the catalytically active Co and Ni species. The authors associated this behaviour with the uniform distribution of multiple metal components, which generated dispersed active sites and lowered the resistance associated with adsorbed reaction intermediates. K_0.8_Na_0.2_(Mg_0.2_Mn_0.2_Fe_0.2_Co_0.2_Ni_0.2_)F_3_, obtained through moderate A-site substitution, further improved the OER performance, exhibiting an overpotential of 314 mV at 10 mA cm^−2^ and a Tafel slope of 55 mV dec^−1^. Na incorporation decreased both the charge-transfer resistance and intermediate-adsorption resistance, thereby facilitating electron transfer and the transport of adsorbed intermediates during oxygen evolution. Wang *et al.* reported a related strategy for OER in alkaline media, in which high-entropy perovskite oxides and fluorides were integrated into multicationic and multianionic oxide-fluoride solid solutions through mechanochemical synthesis.^107^ When Ba_0.5_Sr_0.5_Co_0.8_Fe_0.2_O_3−*δ*_ (BSCF) was used as the oxide component, the resulting (BSCF)_3/4_ [KM(ii)F_3_]_1/4_ solid solution exhibited an overpotential of 345 mV at 10 mA cm^−2^ and operated at approximately 330 mV during a 20 h test (Fig. 10a and b). The high configurational entropy arising from the multiple cations, mixed valence states, and oxide–fluoride anions was proposed to favour the formation of a compositionally mixed single phase, while electrochemical impedance analysis showed reduced resistance associated with adsorbed OER intermediates. The catalytic potential of this multication fluoride system was further demonstrated in nitrate reduction reaction using K(Cu_0.2_Mg_0.2_Co_0.2_Zn_0.2_Ni_0.2_)F_3_ nanocubes by Xue *et al.*^108^ In this case, the high-entropy composition provided multiple metal sites, while morphological control increased their accessibility and improved interfacial transport. Compared with irregular particles of the same nominal composition, the nanocubes exhibited a larger electrochemically active surface area, lower charge-transfer resistance, and a more favourable surface potential, achieving an NH_3_ yield rate of 7.031 mg h^−1^ mg_cat._^−1^ and a faradaic efficiency of 92.8%. The catalyst also maintained a stable current density over 15 h without detectable changes in its crystal structure, nanocubic morphology, or metal valence states, demonstrating the robustness of the multication fluoride framework under nitrate reduction conditions.

The influence of high-entropy design on electronic structure and redox chemistry becomes more apparent in electrochemical energy-storage systems. In lithium-oxygen batteries, Li *et al.* employed K(Co_0.2_Mn_0.2_Ni_0.2_Mg_0.2_Zn_0.2_)F_3_ as an oxygen redox catalyst.^109^ DFT calculations indicated that the multication environment redistributed the transition-metal 3d states, enabling moderate interactions with LiO_2_ intermediates and lower reaction barriers than those single-metal fluorides (Fig. 10c and d). The chemically distinct metal sites also promoted uniform Li_2_O_2_ nucleation, improved interfacial transport, and suppressed electrode passivation and accumulation of Li_2_CO_3_, LiOH, and organic side products during prolonged cycling. The resulting battery delivered a discharge capacity of 22 104 mAh g^−1^ at 200 mA g^−1^ and operated for 500 cycles at 1000 mA g^−1^ under a capacity limit of 1000 mAh g^−1^. High-entropy perovskite fluorides have also served directly as lithium-storage electrodes. K(Mg_0.2_Mn_0.2_Fe_0.2_Co_0.2_Ni_0.2_)F_3_ reported by Wang *et al.* outperformed the corresponding four-component compositions, retaining 120 mAh g^−1^ after 1000 cycles at 2 A g^−1^.^110^ The authors attributed this improvement to entropy stabilization, which buffered structural and volume changes during repeated lithiation and delithiation, together with the complementary functions of multiple redox centres. Ni, Fe, and Mn species participated in conversion reactions, whereas the remaining relatively inactive components helped preserve part of the perovskite fluoride matrix and confine the resulting metallic species. K deficiency additionally provided possible Li-storage sites, while the high-entropy electrode exhibited lower charge-transfer resistance than all of the four-component controls. Su *et al.* later identified more specific transport-related effects in single-phase K_0.9_(Mg_0.2_Mn_0.2_Co_0.2_Ni_0.2_Cu_0.2_)F_2.9_.^111^ The authors proposed that increasing entropy favoured the formation of vacancy-related defects, thereby increasing the BET surface area, opening additional ion-transport pathways, and accelerating Li^+^ diffusion. The electrode delivered 371 mAh g^−1^ after 800 cycles at 2 A g^−1^ and capacities of 698–311 mAh g^−1^ over current densities of 0.1–3.2 A g^−1^. A systematic comparison of low-, medium-, and high-entropy NaMF_3_ compositions by Wang *et al.* further linked configurational complexity to intrinsic electronic transport.^112^ For Na(Mg_0.2_Mn_0.2_Fe_0.2_Co_0.2_Ni_0.2_)F_3_, DFT calculations indicated that transition-metal d-electron delocalization caused the density of states for both spin channels to cross the Fermi level, producing calculated metallic electronic character. Increasing entropy was also accompanied by unit-cell expansion, abundant active sites, faster Li^+^ transport, and a greater pseudocapacitive contribution. These effects supported a capacity of 571 mAh g^−1^ at 0.1 A g^−1^ and retention of 165 mAh g^−1^ after 1000 cycles at 2 A g^−1^. Mechanistic analysis of K(Mg_0.2_Cu_0.2_Ni_0.2_Co_0.2_Mn_0.2_)F_3_ additionally showed how the multiple B-site components participate in conversion-alloying chemistry.^113^ Initial lithiation caused K–F and M–F bond cleavage and generated LiF and metallic species, after which several reduced metal components participated in partially reversible Li-M alloying. This multimetal reaction supplied additional capacity but also caused substantial irreversible lithium consumption. A controlled pre-lithiation strategy was therefore used to establish the conversion products in advance, increasing the initial coulombic efficiency from 36.44% to 119.38% and the maximum specific capacity at 2 A g^−1^ from 587 to 782 mAh g^−1^.

In addition to lithium-based systems, multicomponent perovskite fluorides have been investigated as cathodes for aqueous Zn-based and potassium-ion batteries. Li *et al.* examined a Ni–Co–Zn–Mn fluoride containing partial K/Na mixing at the A site.^114^ The optimized composition with a K/Na ratio of 3 : 1 showed a higher pseudocapacitive contribution, improved OH^−^ adsorption, and faster charge transfer than the K- and Na-only compositions. During cycling in alkaline media, the crystalline fluoride underwent bulk conversion into amorphous metal oxides, hydroxides, and oxyhydroxides that served as the principal redox-active phases. The resulting Zn-based battery delivered energy densities of 110.26–19.90 Wh kg^−1^ over power densities of 1.64–23.11 kW kg^−1^ and operated for 5000 cycles at 8 A g^−1^. Wang *et al.* similarly compared perovskite fluorides containing four, five, and six B-site metal components.^115^ The multicomponent design combined the high redox activity of Ni, the redox diversity and cycling stability of Co and Mn, and the conductivity-enhancing role of Zn (Fig. 10e). The six-component fluoride was converted from ABF_3_ nanocrystals into amorphous mixed-metal oxide and (oxy)hydroxide nanosheets, with reversible charge storage dominated by Ni, Co, and Mn redox reactions. The converted phases exhibited predominantly pseudocapacitive kinetics, while redox electrolyte additives provided additional capacity and enabled flexible devices that remained operational for 10 000 cycles. These aqueous Zn-based systems show that multicomponent perovskite fluorides can function as compositionally controlled precursors to electrochemically generated redox-active phases. For potassium-ion batteries, Liao *et al.* showed that compositionally homogeneous K(Mg_0.2_Mn_0.2_Fe_0.2_Co_0.2_Ni_0.2_)F_3_ nanoparticles embedded in carbon nanofibers suppressed extensive fluoride conversion and instead underwent a highly reversible, low-strain topological intercalation process.^116^ DFT calculations indicated that electrochemically inactive Mg^2+^ increased the free-energy requirement for conversion from 0.17 eV for KFeF_3_ to 1.48 eV, while compositional complexity lowered the K^+^ migration barrier and band gap. The high-entropy fluoride consequently exhibited an electronic conductivity approximately two orders of magnitude higher than that of KFeF_3_, delivered 122 mAh g^−1^ at 20 mA g^−1^, and maintained cycling for more than 5000 cycles at 500 mA g^−1^.

Collectively, these studies show that compositional complexity in perovskite fluorides can regulate electrochemical behaviour through application-dependent pathways. In electrocatalysis, multication mixing can stabilize single-phase fluorides, diversify local adsorption environments, and modify electronic states and the transport of surface intermediates. In battery systems, it can either facilitate the formation of redox-active mixed-metal conversion products or suppress conversion and preserve the fluoride framework, depending on the cation combination and electrochemical environment. Redox-active and relatively inactive cations can therefore play complementary roles in controlling activity, conductivity, ion transport, and structural stability. However, these improvements can not be attributed to configurational entropy alone, because vacancies, lattice dimensions, morphology, particle size, electrolyte chemistry, and conductive architecture also contribute.

## 6. Conclusion and prospect

### 6.1 Core findings and research progress

The main progress in high-entropy perovskite halides is that configurational complexity has moved beyond simple phase stabilization and has become a practical tool for tuning optoelectronic and electrochemical functions. In emissive systems, the best results show that disorder can be beneficial when it is compositionally controlled rather than random. For example, Cs(PbSnSrMnZn)Br_3_ delivers 525 nm emission with a near-unity PLQY of ≈98%, showing that multication alloying can preserve highly efficient radiative recombination while also improving colloidal stability.^39^ A similar trend appears in the red-emitting nanocrystal GA_0.28_Cs_0.72_Pb_0.40_Ge_0.55_Cd_0.05_I_3_, which exhibits 632 nm pure-red emission with an almost near-unity PLQY of ≈100%, demonstrating that entropy-assisted alloying can also support highly efficient and spectrally pure confined emitters.^73^ In blue PeLEDs, where wide-bandgap mixed-halide compositions are especially vulnerable to compositional inhomogeneity and spectral drift, Cs(FA_0.2_Rb_0.1_)Pb(Br_0.6_Cl_0.4_)_3_ provides a representative example with emission at 477 nm and 37% PLQY, while the main text further highlights blue entropy-engineered devices reaching EQEs of ∼11% at ∼477 nm and ∼5.5% at ∼467 nm, indicating that cation disorder is particularly effective in stabilizing radiative recombination in this difficult spectral region.^81^

A central finding in solar cells is that multicomponent perovskites can improve photovoltaic performance by jointly reducing non-radiative recombination, stabilizing the mixed phase, and preserving efficient charge extraction. The strongest experimental example discussed here is (FA_0.98_MA_0.02_)_0.95_Cs_0.05_Pb(I_0.95_Br_0.05_)_3_, which reaches a PCE of 25.7% with *V*_oc_ = 1.17 V, *J*_sc_ = 25.8 mA cm^−2^, and FF = 85.2%, showing that entropy-related compositional complexity can reduce non-radiative loss while maintaining efficient charge extraction.^4^ At the same time, computationally designed B-site high-entropy systems such as Cs_2_(Zr_0.18_Sn_0.36_Te_0.27_Hf_0.09_Re_0.05_Pt_0.05_)Cl_6_ extend this concept further, combining a 1.7 eV bandgap with a predicted PCE of 30.35%, while the related Cs_2_(Zr_0.18_Sn_0.36_Te_0.27_Hf_0.09_Pt_0.1_)Cl_6_ gives 17.67%, demonstrating that the functional value of entropy depends strongly on composition ratio rather than disorder alone.^92^

An important expansion of the field has occurred in high-entropy perovskite fluorides, which extend multicomponent halide design beyond optoelectronics into electrochemical energy conversion and storage. In alkaline OER, K_0.8_Na_0.2_(Mg_0.2_Mn_0.2_Fe_0.2_Co_0.2_Ni_0.2_)F_3_ achieved an overpotential of 314 mV at 10 mA cm^−2^ and a Tafel slope of 55 mV dec^−1^.^106^ The improvement associated with partial A-site substitution was accompanied by lower charge-transfer and intermediate-adsorption resistances, illustrating how A-site regulation can complement a compositionally complex B-site framework. High-entropy perovskite fluorides have subsequently been used as catalysts for OER, nitrate reduction, and lithium–oxygen reactions, as well as electrodes for lithium-, zinc-, and potassium-based energy storage systems.^113–116^ Across these applications, multication environments redistribute transition-metal electronic states, diversify adsorption and redox sites, and allow redox-active and relatively inactive cations to perform complementary functions. Depending on the composition and electrochemical environment, the fluoride framework can either undergo conversion into redox-active mixed-metal oxides and (oxy)hydroxides or suppress extensive conversion and support low-strain ion intercalation. High-entropy design in perovskite fluorides therefore provides control not only over single-phase formation, but also over adsorption, electronic conductivity, ion transport, redox pathways, and structural evolution under operating conditions.

**Fig. 11:**
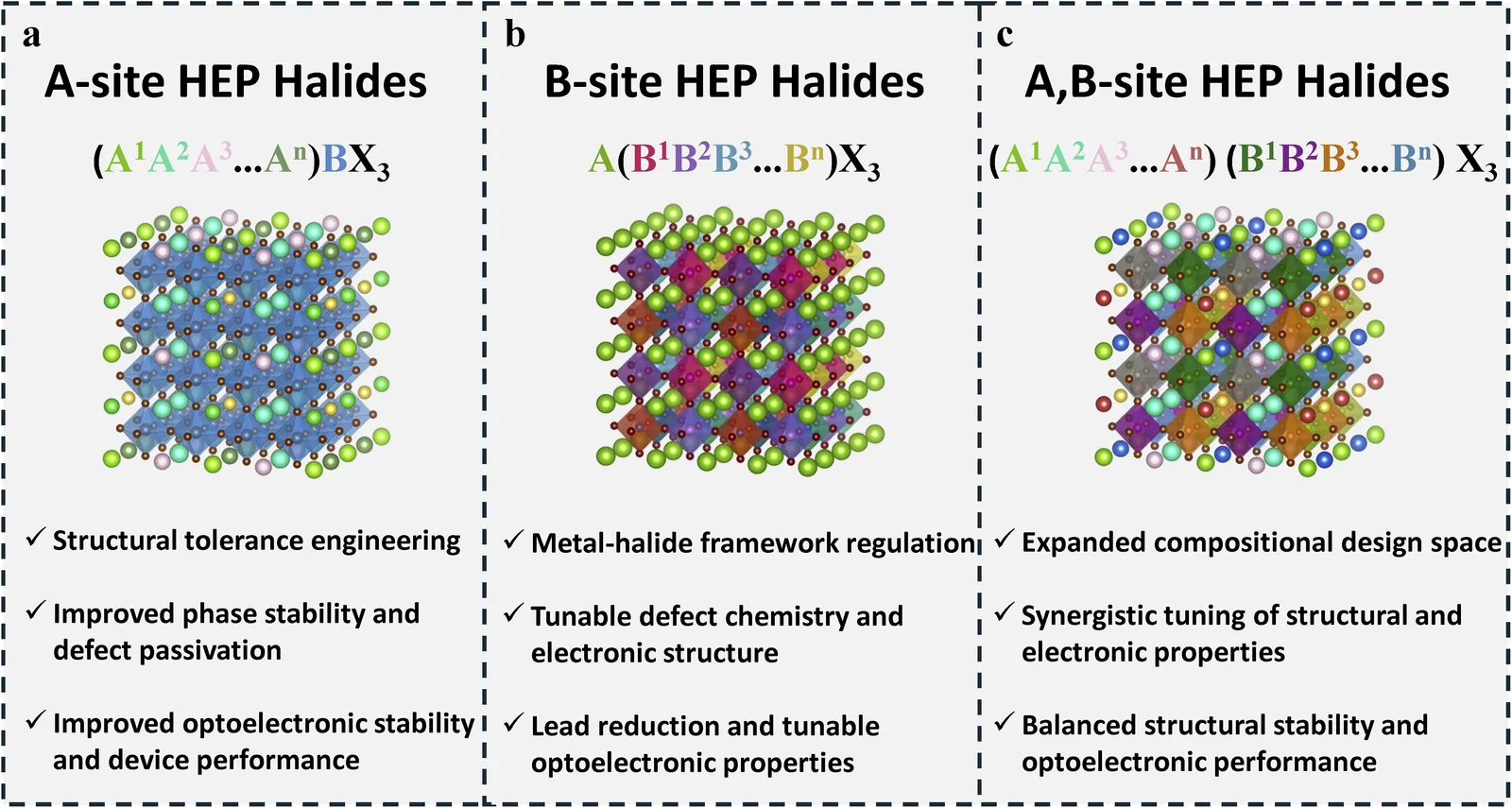
Classification of high-entropy perovskite halides by the site of compositional disorder: (a) A-site, (b) B-site, and (c) A, B-site high-entropy perovskite halides.

Overall, current research indicates that the significance of high-entropy design lies not in maximizing disorder itself, but in using multicomponent complexity as a controllable materials parameter. In practice, this strategy has been realized through three principal routes: A-site engineering, which primarily regulates structural tolerance and phase stability (Fig. 11a); B-site engineering, which more directly modifies the metal-halide framework to tune defect chemistry, electronic structure, and even Pb content (Fig. 11b); and simultaneous A/B-site engineering, which combines these effects to provide the broadest compositional space for coupled structural and optoelectronic control (Fig. 11c). When introduced in a balanced and site-selective manner, configurational complexity can suppress traps, reduce non-radiative losses, stabilize difficult emissive and photoactive phases, and reshape electronic structure in ways that enable high luminescence efficiency, improved spectral stability, and competitive photovoltaic performance. The field has therefore progressed from demonstrating entropy-stabilized perovskite formation to showing that compositional complexity, when rationally designed across different crystallographic sites, can serve as an integrated strategy for simultaneously controlling stability and function in halide-perovskite optoelectronics.

### 6.2 Challenges and outlook

Despite the demonstrated ability of configurational entropy to stabilize perovskite halide lattices, the central challenge in high-entropy systems lies in the mismatch between thermodynamic stabilization and functional optoelectronic performance. While entropy-driven mixing lowers the Gibbs free energy and promotes single-phase formation, this stabilization does not inherently translate into improved electronic structure or optical response. In multicomponent systems, equimolar high-entropy compositions often exhibit irregular and fluctuating absorption behaviours, indicating that excessive compositional disorder can disrupt band-edge states rather than enhance them. Instead, device-relevant properties are highly sensitive to compositional ratios. For example, in Zr–Sn-based systems, an optimized non-equimolar composition yields a bandgap of ∼1.7 eV with balanced carrier effective masses, while further compositional tuning reduces the bandgap to ∼1.5 eV and enhances absorption. Extending this strategy to multicomponent systems leads to significantly improved simulated efficiencies, reaching up to ∼30%, demonstrating that ratio-specific compositional control, rather than entropy maximization, governs performance.^25^

These composition-dependent effects also extend to carrier localization and self-trapped exciton formation, whose influence varies according to the required device function. In scintillators and optically excited phosphors, localization can suppress exciton migration to non-radiative quenching sites and promote radiative emission because long-range charge transport is not required. In photovoltaic devices, however, strong carrier confinement or self-trapping can impede exciton dissociation, carrier diffusion, and charge extraction. High-entropy photovoltaic absorbers should therefore employ site-selective disorder that improves phase stability and suppresses defects while preserving the connectivity and delocalized band-edge states of the inorganic framework. High-entropy hybrid perovskites, in which compositional disorder is accommodated mainly within the organic sublattice while the inorganic framework remains ordered, demonstrate this principle by maintaining efficient charge extraction and achieving a PCE of 25.7%.^4^ In electrically driven PeLEDs, shallow localization or moderate nanoscale confinement may enhance electron–hole overlap and suppress carrier leakage, whereas deep self-trapping or excessive localization can reduce carrier mobility and disrupt charge balance. The optimum degree and location of disorder must therefore be tailored according to whether the application benefits from localized radiative recombination or requires long-range carrier transport.

Identifying these application-specific composition windows is further complicated by the vast compositional space inherent to high-entropy materials. As the number of constituent elements increases, the number of possible compositions grows combinatorially, rendering systematic experimental exploration impractical. Consequently, the primary bottleneck shifts from achieving structural stability to identifying optimal compositions within an effectively unbounded design space. At the same time, the intrinsic instability of perovskite halides remains unresolved. Ion migration continues to dominate structural evolution, with halide ions exhibiting activation energies as low as 0.08–0.58 eV, enabling rapid redistribution under illumination, thermal stress, or electric bias. This results in phase segregation, defect formation, and time-dependent degradation, as evidenced by the emergence of additional photoluminescence peaks near ∼1.68 eV corresponding to compositionally distinct domains.^41^

Attempts to suppress ion migration through multicomponent incorporation introduce an additional trade-off. While multi-element doping can reduce iodide diffusivity by several orders of magnitude and increase migration barriers by ∼15–20%, thereby improving lattice stability, these approaches also increase synthetic complexity and the likelihood of compositional inhomogeneity. As a result, strategies that stabilize the structure simultaneously make precise compositional control more difficult. Taken together, these observations indicate that the key challenge in high-entropy perovskite halides is not simply instability, but the difficulty of simultaneously controlling compositional distribution, ion migration, and electronic structure within a highly coupled system.

Addressing this challenge requires moving beyond empirical compositional exploration toward more guided design strategies. In particular, the vast compositional space of high-entropy systems necessitates data-driven approaches. ML-assisted screening has already demonstrated the ability to identify high-performance compositions from millions of candidates, achieving energy densities up to 20.7 J cm^−3^ with efficiencies of 86% in high-entropy perovskite systems.^62^ More broadly, ML has been applied to correlate compositional, structural, and processing parameters with device performance, enabling more efficient optimization compared to conventional trial-and-error approaches.

From a process perspective, maintaining reproducibility and identifying optimal fabrication conditions remain equally critical. Active-learning-based optimization methods, such as Bayesian optimization, have been shown to converge toward optimal processing conditions with relatively small experimental budgets, typically on the order of 10–100 experiments, significantly accelerating materials and device optimization. In addition, integrating data-driven models with experimental feedback offers a pathway to identify degradation mechanisms and improve long-term stability, although such approaches remain under active development.

Overall, current evidence suggests that progress in high-entropy perovskite halides will depend on combining compositional design, process optimization, and data-driven modelling within an iterative framework. More importantly, future work should focus not on indiscriminately increasing compositional complexity, but on selecting component combinations and composition ratios that preserve structural homogeneity, suppress ion migration, and achieve the desired optoelectronic response.

## Author contributions

R. Levan: conceptualization, visualization, writing – original draft; S. J. Lee: conceptualization, visualization, writing – original draft; T. Bhoyar: conceptualization, visualization, writing – review & editing; K. Lee: funding acquisition, supervision, writing – review & editing.

## Conflicts of interest

There are no conflicts to declare.

## Data Availability

No primary research results, software or code have been included and no new data were generated or analysed as part of this review.
